# ASVBM: Structural variant benchmarking with local joint analysis for multiple callsets

**DOI:** 10.1016/j.csbj.2025.06.045

**Published:** 2025-06-29

**Authors:** Peizheng Mu, Xiangyan Feng, Lanxin Tong, Jie Huang, Chaoqun Zhu, Fei Wang, Wei Quan, Yuanjun Ma, Yucui Dong, Xiao Zhu

**Affiliations:** a School of Computer and Control Engineering, Yantai University, Yantai, Shandong 264005, China; b Department of Hematology, Yantai Yuhuangding Hospital Affiliated to Qingdao University, Yantai, Shandong 264009, China; c Guangzhou Dublin International College of Life Sciences and Technology (GDIC), South China Agricultural University, Guangzhou, Guangdong 510642, China; d Department of Immunology, Binzhou Medical University, Yantai, Shandong 264003, China

**Keywords:** SV benchmarking, SV matching, Joint analysis, Structural variant

## Abstract

Accurate benchmarking of structural variant (SV) detection is essential for advancing the development and application of human whole-genome sequencing (WGS). A fundamental challenge in benchmarking SV detection results is determining whether two SVs represent the same event. Differences in the variation-awareness and strategic implementation of aligners inherently constrain SV detection algorithms that rely on alignment-based approaches. Traditional benchmarking, which primarily focuses on comparing and matching individual variants, makes it difficult to capture the relationships between multiple adjacent variants. We introduced ASVBM, an improved benchmarking framework that introduces the notion of latent positives and leverages a joint analysis and validation strategy based on local variants. This performance improvement arose from the discovery that multiple smaller variants are nearly equivalent to a larger variant. We comprehensively evaluated the performance of six state-of-the-art variant calling pipelines using real WGS datasets. According to multiple matching criteria, ASVBM employs a joint analysis strategy to uncover potential equivalences between the callset and the benchmark set, thereby reducing false mismatches caused by differences in variant representation. ASVBM is available at https://github.com/zhuxiao/asvbm.

## Graphical Abstract

**Figure:**
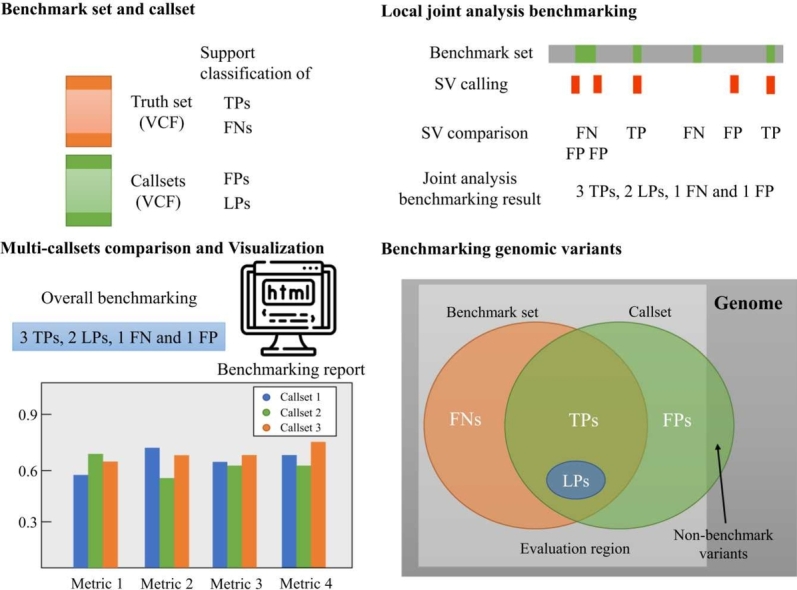

## 1 Introduction

Structural variants (SVs) are major contributors to human genetic diversity and are closely linked to cancer genetics, rare diseases, and evolutionary genetics[1], [2], [3]. Although in human genomics, SVs, including insertions, deletions, duplications, inversions, translocations, and some complex variants (inverted duplications, inverted deletions, etc.)[4], are less frequent than single-nucleotide polymorphisms (SNPs), their larger size allows for the alteration of more bases. SVs can lead to the loss, gain, or reshuffling of genes and regulatory elements, thereby exerting functional impacts that may contribute to both Mendelian and complex diseases. Over the past two decades, significant advancements in SV analysis have not only deepened our understanding of genomic variations but also driven the standardization of SV benchmarking.

Over the past decade, the emergence of long-read single-molecule sequencing technologies from Pacific Biosciences (PacBio) and Oxford Nanopore Technologies (ONT) has revolutionized sequencing capabilities[5], [6], [7], [8]. These technologies generate average read lengths of 20 kilobase pairs (kbp) or higher, enabling the resolution of variation in longer and more complex repetitive DNA, thereby increasing the complexity of the human genome[9]. Long reads are also more likely to span SV breakpoints, providing high-confidence alignments. Long reads offer improved accuracy and reliability in detecting large SVs compared to short-read sequencing, driving the development of diverse SV identification algorithms[10], [11], [12], [13], [14]. These improvements primarily focused on SV discovery and genotyping within the context of single samples. Concurrently, SV benchmarking standards established by organizations such as the Genome in a Bottle Consortium (GIAB) have provided an objective measure of SV detection results[15]. However, the issue of identifying matching SVs remains inadequately addressed.

A significant challenge in comparing two variant call format (VCF) files is the accurate handling of complex variant representations. In VCF files, variant calling does not always correspond to the same haplotype sequence. The drawback of traditional long reads is their error-prone sequencing[16]. Long reads have significantly lower per-base accuracy than short-read data, with raw basecalling errors of 3–15 %[17]. Although HiFi or circular consensus sequencing (CCS) PacBio and Nanopore reads generated using the latest flowcells R10.3 have lower error rates, their error rates can still be significantly higher than those of short-read data[18]. Even with standardized gap penalties and substitution scoring, sequence alignments may not be deterministic, and different variant callers may produce different representations of the same variant. Although certain discrepancies can be mitigated through preprocessing steps such as left alignment and variant trimming, others are inherently difficult to resolve[19]. Furthermore, the absence of phasing information in the majority of VCF files generated by existing variant calling pipelines exacerbates these challenges. Consequently, relying solely on the comparison of individual SV records is insufficient for a precise benchmarking of VCF files.

Since numerous SVs are located within tandem repeat regions, coupled with the inherent imprecision in SV calling and the absence of standardized representations for various types of complex SVs, benchmarking these variants presents even greater challenges[20]. Furthermore, the accuracy of SVs can be benchmarked at varying levels of stringency. For instance, "low stringency" may only require correct identification of the SV type and approximate location, such as SURVIVOR[21], whereas "high stringency" requires precise sequence and accurate annotation, such as Truvari[22]. For small variants, tools such as hap.py[19] employ a strict haplotype matching mechanism. This mechanism treats adjacent variations as a group, requiring all variants within the group to be accurately detected, thereby ensuring the accuracy of haplotype transmission. However, this method is unsuitable for large-scale SVs. Isolated insertions and deletions within non-repetitive sequences are typically the most straightforward to detect and benchmark, yet they constitute only a minor subset of all SVs[15], [23]. Although phased long-read technologies have enhanced the resolution of complex SVs, advanced benchmarking tools, such as Truvari and hap-eval still face significant challenges in comparing diverse SV representations[20], especially for non-simple insertions or deletions.

A key challenge in SV benchmarking is establishing robust matching criteria. SVs are identified using different sequencing experiments or heterogeneous pipelines may exhibit discrepancies in location and sequence due to variations in pipeline sensitivity[24], alignment ambiguities near repetitive regions[25], or inherent differences in the algorithms[26]. Any combination of these factors can impact the benchmarking outcome. Overly lenient matching can cause incorrect consideration of adjacent SVs as matches, whereas excessively stringent criteria may fail to identify accurate matches, ultimately compromising the objectivity of benchmarking results.

Recently, multiple strategies have been proposed for SV matching. One strategy relies on breakpoint consistency, where two SVs are considered to match if their breakpoints fall within a predefined range (500–1000 base pairs (bp))[22]. This method is more suitable for larger SVs but tends to be less sensitive for smaller ones (<100 bp). Alternatively, SVs with matching breakpoint orientations at both breakpoint junctions and located within 50 bp of each other can be considered identical[26]. However, these approaches are limited by discrepancies in aligners. Reciprocal overlap[27] is beneficial for matching similar SVs (copy number variants in the same gene) with imprecise breakpoints[28], but it is inapplicable for insertions lacking a reference sequence span. Furthermore, the presence of multi-allelic variants has introduced complexity into benchmark datasets, as sequence variations can occur even at the same location. Given the potential sequence differences between alleles, it is crucial to comprehensively consider variant characteristics when matching allelic loci. Extending the upstream and downstream sequences from the reference for comparison is common to improve matching accuracy. However, the same variant may be placed in different positions due to differences in aligners, especially in complex genomic regions such as those with repetitive sequences. Additionally, for small indels, differences in variant positions may cause excessive extension of non-variant fragments, which can substantially increase the length of the compared sequence relative to the original variant sequence and potentially affect the precision of variant matching. Individually, each of these approaches may mistakenly categorize SVs as matching.

Comparisons cannot fully address the challenges posed by binary classification models due to the inherent complexity of the human genome. Existing aligners typically employ locally optimal matching strategies, which tend to overlook the global context of variants. Consequently, larger SVs are sometimes represented as combinations of multiple smaller variants. To address the issues of high error rates inherent to long-read sequencing data and alignment inaccuracies, we introduced ASVBM, an improved SV benchmarking method that incorporates the concept of latent positive (LP) and employs a local joint analysis and validation approach for variants, compensating for the limitations of traditional benchmarking methods in reducing false positive (FP) and false negative (FN) arising from the representation of multiple smaller variants. We benchmarked the performance of various SV callers on the HG002 CCS[29] dataset using ASVBM. ASVBM consistently yielded results comparable to those of three other benchmarking methods. We assessed their overall effectiveness in SV detection by analyzing the SV calls generated by each tool. Compared to other methods, ASVBM employs a joint analysis approach to detect potential mismatches caused by differences in variant representations while maintaining broad usability.

## 2 Materials and methods

### 2.1 Datasets

HG002 PacBio CCS data and PacBio Continuous Long Reads (CLR) data used in this study were downloaded from the National Center for Biotechnology Information (NCBI) with accession numbers SRX5327410 and SRX7668835. The datasets are formatted through SRA Toolkit (https://github.com/ncbi/sra-tools). SeqKit[30] was used to extract sequencing data with 50 × coverage from SRX7668835. A total of 39 PacBio Sequel sequencing runs were performed, generating approximately 6.6 million sequencing reads with average read length 13.5 kbp and mean coverage depth of approximately 30x. The high-confidence benchmark set for HG002, consisting of a total of 74,012 variants including 37,412 deletions and 36,600 insertions, was used as the ground truth. This benchmark set was developed by GIAB and is available at ftp://ftp-trace.ncbi.nlm.nih.gov/giab/ftp/data/AshkenazimTrio/analysis/NIST_SVs_Integration_v0.6. The human reference genome GRCh37 was downloaded from the International Genome Sample Resource (IGSR)[31] FTP server at ftp://ftp.1000genomes.ebi.ac.uk/vol1/ftp/technical/reference/phase2_reference_assembly_sequence/hs37d5.fa.gz.

### 2.2 Benchmarking SV callers

Reads were mapped using ngmlr (v0.2.7) and minimap2 (v2.24-r1150-dirty) technology-specific preset parameters. The reference genome GRCh37 was used to test for the GIAB v0.6 SV benchmark. The resulting alignments were then converted into the BAM format using samtools[32] (v1.16.1). Subsequently, the BAM files were sorted and indexed using the same samtools software.

To establish a baseline for comparison, SV callers were initially benchmarked using their default parameters, with the exception of the minimum variant size was uniformly set to 20 bp across all tools, consistent with the smallest variant size in the benchmark set. These callers included SVDSS (v2.0.0), Sniffles2 (v2.0.2), and SVIM (v2.0.0). DeBreak (v1.0.2) was configured to utilize additional parameters specifically designed to improve the identification of large insertions, duplications, and complex rearrangements. These additional parameters included --rescue_large_ins, --rescue_dup and --poa. For pbsv (v2.9.0), the --ccs option was included during SV calling to optimize the analysis for HiFi sequence data. cuteSV (v2.0.3) was employed with the additional parameter --genotype to enhance variant calling accuracy. Finally, ASVBM (v1.3.0), Truvari (v4.0.0), and svbench (v0.7.6) were utilized to comprehensively evaluate the identified results from all callers.

### 2.3 Overview of ASVBM

**Fig. 1:**
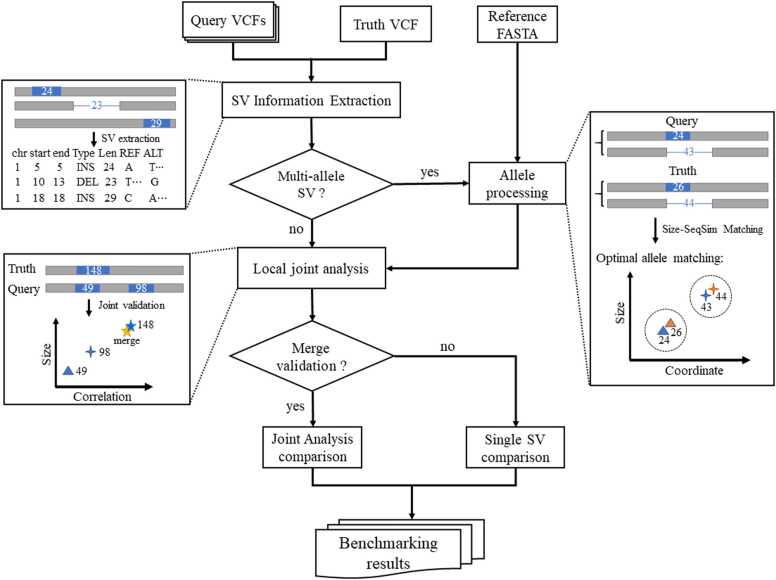
**Workflow of ASVBM's multiple user callsets benchmarking.** The specific steps are as follows: (1) extract the required information for benchmarking from the input user callsets, such as chromosome, position, SV type, REF and ALT sequences; (2) determine alleles: perform matching according to size and sequence similarity to obtain the optimal matches; (3) local variant joint analysis and validation: merge adjacent variants (including multi-allelic SVs) to validate larger SV in the benchmark set; (4) SV comparison: SVs that meet five predefined criteria are considered matched variants and classified as true positives (TPs) or latent positives (LPs). Otherwise, they are categorized as false positives (FPs) or false negatives (FNs); (5) output benchmarking results: generate the benchmark report.

Our primary use case focuses on a benchmark set that can serve as a "ground truth" dataset, such as GIAB or Platinum Genomes, enabling the benchmarking of one or more callsets against this dataset. The inputs for this comparison include a variant callset and a ground truth set in VCF format. Additionally, a reference FASTA file is provided to validate the sequence-resolved accuracy of the callsets. The workflow of ASVBM is presented in Fig. 1. ASVBM performs SV matching between the user and benchmark sets using five criteria: SV type matching, reference distance, overlap, size similarity (e.g., 0.7), and sequence similarity (e.g., 0.7). ASVBM introduces the sequence similarity metric to capture matches between sequence-resolved calls through sequence alignment. These default thresholds, are typically applied to comparisons between a single SV and a standard SV benchmark set and are suitable for high-similarity sequence-resolved calls. However, they can be adjusted according to user specifications. We aimed to develop a standardized framework for the variant benchmarking process with the following goals:

• The methods used to compare identification sets are independent of different representations of the same variant.
• Primary performance metrics are expressed using the widely accepted binary classification format, including true positive (TP), FP, FN, and derived statistics, with the concept of LPs incorporated to address cases in which multiple smaller variants are nearly or entirely equivalent to a single larger variant.
• The calculation of performance metrics follows a standardized approach, enabling more consistent and straightforward comparisons across multiple callsets.
• Performance metrics are stratified by variant size to allow for detailed and meaningful result analysis.

An additional strength of ASVBM is its ability to benchmark multiple user callsets simultaneously, generating an HTML report to visualize benchmark results and creating an upset plot to depict overlaps of TPs.

The distance between the breakpoints of the corresponding SVs in the SV identification results and the benchmark set is calculated to determine the size and location discrepancies between the two sets of SVs. To assess these discrepancies, two additional metrics are employed: breakpoint distance (*d*) and variant size ratio (*r*). Finally, the analysis results are summarized to generate a benchmarking report that can be used to assess the performance of the SV calling pipelines.

### 2.4 Benchmarking method

#### 2.4.1 SV comparison calculation between user set and benchmark set

SV detection results typically encompass a wide range of SV sizes, with SVs < 100 bp constituting a significant proportion of the total, and this proportion steadily decreases as SV length increases[1], [33]. ASVBM extracts variant information from both the user callset and the benchmark set, which are provided in VCF v4.2 format[34] (Supplementary Note 1). SV items with lengths > 50 kbp by default are excluded from further analysis.

The core function of ASVBM is to benchmark SV identification results by comparing them with a reference benchmark set. The accelerated matching method employed by ASVBM involves the following steps (Supplementary Figure S1):

(1) Dividing and sorting: To minimize unnecessary computations across chromosomes, the data are first divided into chromosome-specific subsets. Within each subset, SVs are then sorted by location to create an ordered dataset.
(2) Targeted search space: For each SV in the user callsets, a focused search space is created within the corresponding benchmark set of chromosomes. This space typically spans approximately one-hundred thousandth of the chromosome length.
(3) SV matching: If any records within this search space meet predefined matching criteria (see section "Variant comparison methodology"), the SV and the matching record(s) are considered successful matches.

For SVs ≥100 bp, the same variant records may appear at distinct locations across different datasets due to variations in alignment tools and slight size discrepancies. To address this situation, SVs ≥100 bp undergo further processing: (1) If no match is found within the initial 200 bp search space, the search space is redefined by extending 1 kbp on each side of the variant position, and the search is repeated; (2) SVs found in the expanded space are filtered according to size similarity (ratio between 0.7 and 1.2) and matching SV types. (3) Sequence similarity is considered a part of the benchmarking process for SVs that meet the matching criteria. Initially, sequence similarity is calculated using the variant sequences provided for both variants (Supplementary Figure S2). If the SVs reside at different genomic locations, a shared reference region is defined by extending from the upstream-most start position to the downstream-most end position across both SVs, ensuring that both SVs are evaluated within a comparable genomic context. Subsequently, the Needleman-Wunsch (NW) alignment algorithm is employed to align these two sequences. Then, the sequence similarity is computed and reported based on the alignment. A predefined threshold (default 0.7) is applied to sequence similarity, and the SVs exceeding this threshold are considered to be SV matching. Finally, SVs within the user set that successfully match entries in the benchmark set are designated as TPs.

#### 2.4.2 Allele-aware SV matching

SV matching associated with allelic variants is a critical step in SV detection. This process involves identifying and comparing SVs that represent different allelic forms within individual genomes. The presence of allelic variants introduces complexity to the benchmark sets, which serve as the gold standard for evaluating and validating SV detection methods. This complexity presents a challenge for benchmarking. The SV detection results for allelic variants require matching of each SV record against corresponding entries in the benchmark set (Supplementary Figure S3). ASVBM incorporates an allele matching strategy that accounts for the query's zygosity (homozygous or heterozygous) and corresponding variant in the benchmark set. ASVBM allows comparisons between query variants and benchmark heterozygous variants. When both the query and baseline variants independently meet the matching criteria, ASVBM considers both to be TPs. ASVBM incorporates a sequence similarity-based optimization strategy to further refine the matching, selecting the baseline variant with the closest sequence similarity to the query variant as the optimal match.

#### 2.4.3 Joint analysis of local variant validation

**Fig. 2:**
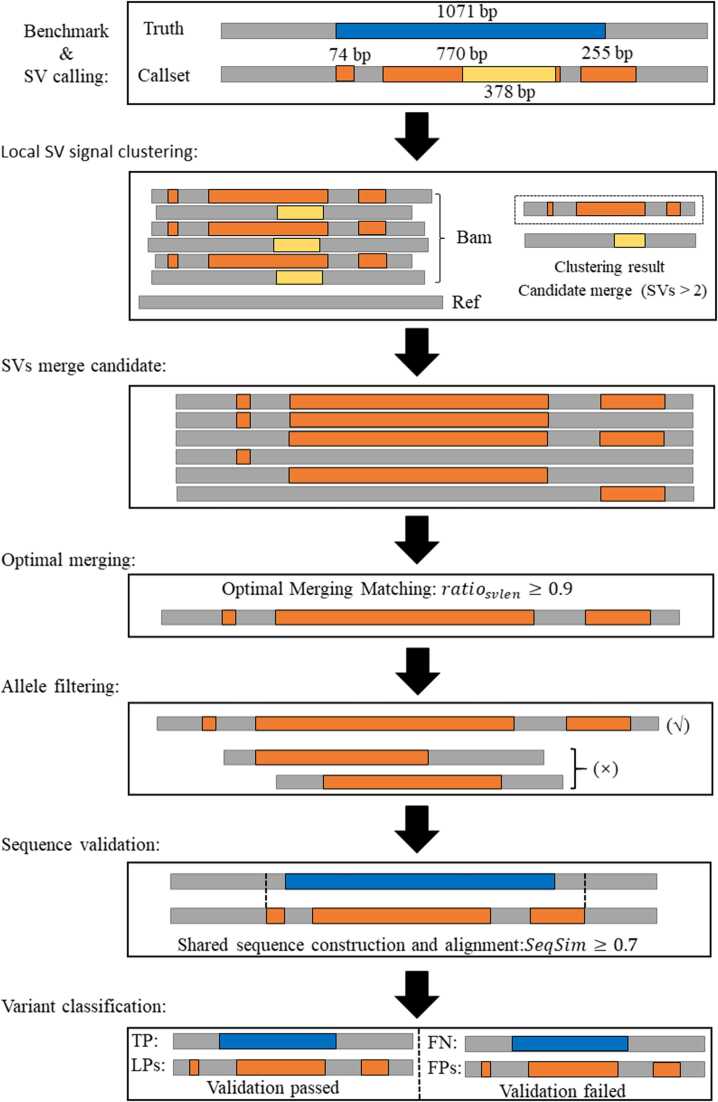
**Local variant joint analysis workflow.** SVs within the distance threshold are first grouped by SV type, with merging candidates further determined based on the original SV signals from the BAM files. Next, an optimal matching strategy, considering variant size, identifies potential merge candidates. Subsequently, the candidate set is filtered by removing alleles, followed by the construction and alignment of shared sequences to evaluate alignment-based consistency between variants. Finally, SVs are classified into two categories according to the validation results.

This benchmarking method introduces the concept of LPs and adopts two distinct matching strategies: (1) single matching, a single variant is matched to a nearby variant within the benchmark set, and (2) local matching, a local subset of variants from the callset is considered a TP if it matches a variant in the benchmark set, and the subset is classified as an LP. TP is an SV record found in the benchmark set and correctly detected by the user callset. LPs are defined as variant calls from the user callset that, when combined or merged with adjacent variants, constitute a representation with a high degree of concordance with a TP in the benchmark set. These variants may individually lack sufficient confidence to match a benchmark variant but achieve equivalence or near-equivalence to a TP when considered in aggregate. Given the combinatorial complexity of exploring all possible variant groupings, particularly in complex genomic regions, this method adopts a heuristic merging strategy based on SV types and breakpoint proximity. Unlike previous benchmarking methods, ASVBM credits partial TPs to the SV caller. Although individual variant calls may not match benchmark variants at the base-pair level, merge-based validation demonstrates their equivalence to benchmark variants (Fig. 2). The first step is to identify the candidate variant set for merging by considering SV signatures, such as breakpoints and SV types, to create superclusters. ASVBM supports input in BAM format and scans the raw SV signals across all read alignments for each SV category, filtering merged candidate variants through haplotype partitioning. The second step is to perform merging validation, where variants within a cluster are sequentially combined based on their breakpoint positions. The lengths of the merged variants must high similarity to the SVs in the benchmark set. To minimize the risk of erroneous multi-allelic variants merging, variants with overlapping original coordinates are filtered. Finally, leveraging the regions covered by the positions of the variants in the merging cluster and those in the benchmark set, upstream and downstream reference are extracted to construct a shared contextual sequence. Variants within the supercluster that satisfy the sequence similarity threshold are classified as LPs, while the corresponding benchmark variant is classified as a TP. Conversely, if no variant within the supercluster meets the sequence similarity threshold, the corresponding benchmark variant is classified as an FN, and variants in the supercluster are classified as FPs. This improvement mitigates the limitations of traditional benchmarking methods, which may result in missed detections of multiple small variants, thereby enhancing the robustness and precision of benchmarking.

#### 2.4.4 Variant comparison methodology

SVs are characterized by several key properties, including start position (*S*), end position (*E*), length (*L*), and variant sequence (*V*). Additionally, SVs possess a corresponding reference sequence (*R*). The *L* of SV is often provided in the VCF file, but when it is missing, it can be calculated using genomic coordinates or sequence content. For deletions, the length (*Len*(*V*)) is calculated as the difference between the *E* and *S*. However, the insertions have no span over the reference, and length is simply *Len*(*R*). To determine whether the two variants overlap, the following approach is employed: both variants are extended by a specific length (*extend_len*) according to their lengths. The NW sequence alignment algorithm is used to align the extended variants for sequence similarity calculation. Notably, the NW algorithm may introduce consecutive gaps within the alignment. ${\mathrm{Alnseq}}_{X}$ and ${\mathrm{Alnseq}}_{A}$ represent the alignment results before and after extracting the reference genome sequences, respectively, based on the variant positions. *G* is defined as the total number of gap characters in gap runs of length three or longer, and *M* represents the total number of matching bases between the variant sequences. A tunable parameter, the relief factor (RF), is introduced to reduce the impact of long-gap runs on the sequence similarity score,. This parameter downweights the negative effect of excessive gaps in the alignment, allowing for more balanced comparisons between variant sequences. Formal definitions of each matching criteria follow:

**Reference distance**: Variant’s positions are within the specified *extend_len*, as determined by the length of the variant:

$$\mathrm{extend}\_\mathrm{len}=\left\{\begin{array}{cc} 200\mathrm{bp}, & L\leq 100\mathrm{bp}\\ 1\mathrm{kb}, & L>100\mathrm{bp} \end{array}\right)$$

**Overlap**: Determine the overlap on the variant span using the extended variant positions:

$$\mathrm{ovL}\_S=\max(\mathrm{extend}\_S_{1},\mathrm{extend}\_S_{2})$$

$$\mathrm{ovL}\_E=\min(\mathrm{extend}\_E_{1},\mathrm{extend}\_E_{2})$$

$$\mathrm{ovl}=\left\{\begin{array}{cc} \mathrm{overlap}, & \mathrm{ovl}\_S\geq \mathrm{ovl}\_E\\ \mathrm{disjoint}, & \mathrm{ovl}\_S<\mathrm{ovl}\_E \end{array}\right)$$

**Size similarity**: Minimum SV size over the maximum SV size:

$$\mathrm{sizeSim}=\left\{\begin{array}{cc} \frac{\min\left(\mathrm{Len}(V_{1}),\mathrm{Len}(V_{2})\right)}{\max(\mathrm{Len}(V_{1}),\mathrm{Len}(V_{2}))}, & \mathrm{SVTYPE}\neq \mathrm{DEL}\\ \frac{\min(\mathrm{Len}(R_{1}),\mathrm{Len}(R_{2}))}{\max(\mathrm{Len}(R_{1}),\mathrm{Len}(R_{2}))}, & \mathrm{SVTYPE}=\mathrm{DEL} \end{array}\right)$$

**Sequence similarity**: Haplotype sequence similarity is calculated using the NW algorithm, taking the optimal score before and after incorporating reference sequence:

$$S=\min\left(S_{1},S_{2}\right)$$

$$E=\left\{\begin{array}{cc} \max\left(S_{1}+{\mathrm{Len}}_{R_{1}}-1,S_{2}+{\mathrm{Len}}_{R_{2}}-1\right), & \mathrm{SVTYPE}=\mathrm{INS or DEL}\\ \max\left(E_{1},E_{2}\right), & \mathrm{SVTYPE}=\mathrm{DUP or INV} \end{array}\right)$$

$$A_{1}=\left\{\begin{array}{cc} \mathrm{Ref}\left[S:S_{1}\right]+V_{1}+\mathrm{Ref}\left[E_{1}:E\right], & \mathrm{SVTYPE}\neq \mathrm{DEL}\\ \mathrm{Ref}\left[S:S_{1}\right]+R_{1}+\mathrm{Ref}\left[E_{1}:E\right], & \mathrm{SVTYPE}=\mathrm{DEL} \end{array}\right)$$

$$A_{2}=\left\{\begin{array}{cc} \mathrm{Ref}\left[S:S_{2}\right]+V_{2}+\mathrm{Ref}\left[E_{2}:E\right], & \mathrm{SVTYPE}\neq \mathrm{DEL}\\ \mathrm{Ref}\left[S:S_{2}\right]+R_{2}+\mathrm{Ref}\left[E_{2}:E\right], & \mathrm{SVTYPE}=\mathrm{DEL} \end{array}\right)$$

$$\left(A\ln{\mathrm{seq}}_{X_{1}},{\mathrm{Alnseq}}_{X_{2}}\right)=\mathrm{NW}\_\mathrm{Algorithm}\left(X_{1},X_{2}\right),X=\left\{\begin{array}{cc} V, & \mathrm{SVTYPE}\neq \mathrm{DEL}\\ R, & \mathrm{SVTYPE}=\mathrm{DEL} \end{array}\right)$$

$$\left(A\ln{\mathrm{seq}}_{A_{1}},{\mathrm{Alnseq}}_{A_{2}}\right)=\mathrm{NW}\_\mathrm{Algorithm}\left(A_{1},A_{2}\right)$$

$$（M_{X},G_{X}）=A\ln\mathrm{Stat}（A\ln{\mathrm{seq}}_{X_{1}},{\mathrm{Alnseq}}_{X_{2}}）$$

$$（M_{A},G_{A}）=A\ln\mathrm{Stat}（A\ln{\mathrm{seq}}_{A_{1}},{\mathrm{Alnseq}}_{A_{2}}）$$

$$\mathrm{SeqSim}=\max\left(\frac{M_{X}+(G_{X}*\mathrm{RF})}{\mathrm{Len}\left({\mathrm{Alnseq}}_{X}\right)},\frac{M_{A}+(G_{A}*\mathrm{RF})}{{\mathrm{Len}(\mathrm{Alnseq}}_{A})}\right)$$

A fixed extension of 100 bp upstream and downstream of the reported breakpoint is applied to enable overlap determination of structural variants that lack a physical span in the reference genome, such as insertions and other complex variants. In sequence similarity calculations, deletions are typically reported in the REF column of the VCFs; therefore, the deletion sequence is derived from the REF column, creating a shared context for calculating similarity. To compute sequence similarity, calculations are performed before and after constructing the shared context with the extracted reference, and the optimal score is taken as the final result (see section “Effective sequence similarity calculation”). The end position of insertions and deletions is determined according to the variant size, as the END in the INFO column of the VCF may sometimes be inaccurate for insertions, and this issue is particularly common in benchmark sets generated from simulated datasets using tools such as SURVIVOR.

#### 2.4.5 Effective sequence similarity calculation

ASVBM calculates sequence similarity based on variant sizes. For tandem duplications and inversions, which are typically non-sequence-resolved, the ALT alleles in VCF are represented as symbolic alleles, denoted by an angle-bracketed ID such as <INV> or <DUP> ). Extracting contextual sequences solely from the reference to calculate sequence similarity has certain limitations for these SVs. A match is considered valid if other criteria are met. We focus only on sequence similarity comparisons of insertions and deletions. For insertions, variant sequences are identified between the benchmark set and the user callset. Subsequently, sequences surrounding the variant site are extracted, and sequence similarity is calculated. However, when multi-allelic variants or single-nucleotide variants (SNVs) are present in the SV region (Supplemental Figure S4), the extended reference sequence may not accurately represent the variant sequences of all reads, which may introduce discrepancies. In these cases, the sequence similarity calculated from the extracted reference sequence may be underestimated due to the presence of SNVs within reads supporting multi-allelic variants or alignment ambiguities. Therefore, taking the maximum of sequence similarity calculated before and after extraction reduces the effect of SNVs, on sequence similarity calculations.

On the other hand, the NW sequence pairwise alignment algorithm may consume a substantial amount of memory during the calculation of sequence similarity, particularly when simultaneously handling multiple large variant sequences (Supplementary Figure S5). Therefore, *k*-mers-based methods are generally used for larger SVs (>10 kbp) where the two sequences are similar. These methods employ a divide-and-rule approach to locate homologous fragments as "anchors" and divide the sequences into multiple subsequences[35]. Additionally, minimizers are used to select a common subset of *k*-mer from similar sequences[36]. ASVBM sets the default *k*-mers length to 15 and the window size to 10. In each window, ASVBM selects the *k*-mers with the minimum hash value as the minimizer, and traverses the entire sequence to construct an ordered set of minimizers. Subsequences are searched based on constraints of sequence and location similarity to capture the similarity between sequences. After calculating the subset of subsequences, redundant subsequences were removed based on their locations in the sequences. Afterward, the hash values of corresponding items in the two common subsequence sets were filtered, retaining only those with matched hash values. Finally, following the principle of proximity in location, the sequences only between the two adjacent minimizers (i.e., homologous sequences) in the two common subsequence sets were aligned. Then, the aligned sequences were merged with homologous sequences to calculate overall sequence similarity.

#### 2.4.6 Fine-grained analysis of the deviation of SV records

For the results of the SV callers[37], [38], [39], [40], [41], [42], [43], [44], [45], [46], there are generally marked discrepancies between certain SV regions in the user callset and the benchmark set. To achieve a more fine-grained assessment of detection result deviation, ASVBM employed two metrics to quantify the discrepancies between identified variants and their corresponding targets within the benchmark set: (1) *breakpoint distance* (*d*); and (2) *variant size ratio* (*r*). Briefly, *d* represents the genomic distance between the predicted breakpoints and the corresponding reference breakpoints, while *r* is defined as the ratio of the length of the predicted variant to the length of reference variant. ASVBM supports calculating these metrics for insertions, deletions, duplications, and inversions. The *breakpoint distance* of SV region [*start_pos*, *end_pos*] is defined as follows:

If the variant *s* overlaps with the variant *t*, the *breakpoint distance* of the two SV regions [*start_pos*_*s*_, *end_pos*_*s*_] and [*start_pos*_*t*_, *end_pos*_*t*_], as well as the *variant size ratio*, are defined as:

$$d={\mathrm{start}\_\mathrm{pos}}_{s}-{\mathrm{start}\_\mathrm{pos}}_{t}$$

$$r=\frac{\mathrm{en}{d\_\mathrm{pos}}_{s}-\mathrm{start}{\_\mathrm{pos}}_{s}+1}{\mathrm{en}{d\_\mathrm{pos}}_{t}-\mathrm{start}{\_\mathrm{pos}}_{t}+1}$$

The closer the *breakpoint distance* is to 0 and the *variant size ratio* is to 1, the smaller the deviation and the more accurate the identification result.

#### 2.4.7 Size-based classification of SVs

The SV identification results typically encompass a wide range of variant sizes. Categorizing these variants into distinct size ranges facilitates a more fine-grained exploration of identification results. This approach offers valuable insights into the sensitivity of SV callers to variants of different sizes. ASVBM provides configurable size-based classification of SVs, with eight default size bins ranging from small (<100 bp) to large (>10 kbp) variants. The user can customize these size categories to suit specific research requirements or dataset properties. This categorization enables a more detailed analysis of caller performance, and for each size category, ASVBM calculates key metrics.

## 3 Results

### 3.1 Performance on real datasets of the HG002 individual

Benchmarking tools vary in their evaluation capabilities, and each is designed to address specific types of variants or analytical tasks, thereby making them suitable for different benchmarking objectives. Specifically, hap.py is primarily used for benchmarking SNPs and indels. However, other tools offer broader capabilities to compare VCF files, address the complex challenges posed by SVs, and enable better handling of complex variants, such as insertions, deletions, and duplications. Additionally, ASVBM and svbench[47] support benchmarking multiple user callsets simultaneously with a single command, simplifying the evaluation process. ASVBM and Truvari calculate the sequence similarity of matched SVs, providing more reliable matching results, but, ASVBM adopts a local joint variant analysis strategy, rather than focusing solely on the comparison of individual SVs. Furthermore, the capacity for visualization and report generation of these tools is essential for comprehensively understanding the benchmarking results. The core features and characteristics of these methods are summarized in Table 1. More detailed benchmarking results can be obtained using ASVBM.

**Table 1:** Functional comparison of tools for structural variant benchmarking.

Features	ASVBM	Truvari	svbench	hap.py
Input format	vcf; ref	vcf.gz; [ref]	vcf	vcf.gz; ref
Variant type	Indel; SV	Indel; SV	Indel; SV	SNP; Indel
Classification stat	✓	✓	✗	✓
Multiple callsets	✓	✗	✓	✗
Multithreading	✓	✗	✗	✗
Allele-aware	✓	✓	✗	✓
Joint analysis	✓	✗	✗	✗
Genotype	✗	✓	✓	✓
Sequence similarity	✓	✓	✗	✗
DUP/INS match	✓	✓	✗	✗
Reported metrics	TP; FP; FN; LP	TP_base; TP_comp; FP; FN	TP; FP; FN	TP; FP; FN
Size distribution	✓	✗	✗	✗
Range statistics	✓	✗	✓	✗
Visualization	HTML; Upset; Text	vcf.gz; json	Text	vcf.gz; csv.gz

Note: Comparison of features supported by different benchmarking tools. Sequence similarity refers to the score that measures the similarity between SV comparison records. ASVBM calculates both the sequence similarity for the SV comparison records as well as the average sequence similarity across all matched SVs, whereas Truvari only reports the sequence similarity for the SV comparison records. Reported metrics represents the categories of metrics used for statistical evaluation. Size distribution refers to the distribution of SV counts. Range statistics refers to dividing SVs into multiple bins based on their magnitudes and counting the number of variants in each bin. Its purpose is to conduct multi-bin evaluations of structural variations. A check mark (✓) indicates the presence of a feature in the tool, ✗ indicates not applicable, and content within brackets ([]) represents optional parameters.

A common approach for benchmarking is to establish a ground truth by reaching a consensus between calls from various sequencing technologies and SV discovery algorithms and then comparing new calls to this ground truth. In this experiment, human HG002 PacBio CCS sequencing data were used for SV calling. The 2020 GIAB v0.6 callset was employed as the benchmark due to its established reliability and comprehensiveness, specifically including variants longer than 20 bp. The HG002 CCS data were aligned to the GRCh37 reference genome using minimap2[48]. To ensure state-of-the-art performance, the following SV detection methods were chosen for benchmarking: SVDSS[37], pbsv (https://github.com/PacificBiosciences/pbsv), DeBreak[38], Sniffles2[39], cuteSV[40], and SVIM[41]. All methods were run with a minimum SV length of 20 bp to ensure consistency with the benchmark set. This comprehensive benchmarking approach allows for a thorough analysis and comparison of various SV calling methods.

we compared our method to two widely used tools for validating SV calls, Truvari and svbench, using the HG002 human genome sample. These methods, along with ASVBM, were compared on callsets generated by the six SV discovery methods, including SVDSS, DeBreak, Sniffles2, pbsv, cuteSV, and SVIM. Since the benchmark set only includes insertion and deletion variants, other types were excluded from in the comparison, except duplications. Due to the inability of svbench to specify chromosomes, coupled with the inclusion of decoy sequence variants in the variant detection results, the precision and F1 score of svbench's evaluations were lower. We demonstrated that within regions annotated as high confidence by GIAB, ASVBM produced consistent results with Truvari using the GIAB gold standard callset (Table 2). Minor differences in the implementation of benchmark tools such as Truvari, and svbench have a significant impact on the results. Currently, there are significant differences in how each tool handles correctly identified variants. ASVBM, which employs a local joint analysis validation method, matched more TPs due to its LP metrics. It more effectively addresses partially correct but not entirely correct variants, which are prone to be misclassified as FPs or FNs, thereby better overcoming the limitations of traditional benchmarking that only compares and matches individual variants.

**Table 2:** Benchmarking results on the GIAB SV dataset for HG002 on Multiple callsets.

	software	**#**TP	**#**FP	**#**FN	#LP	Rc	Pr	F1	SeqSim	Real time (sec)	Peak Mem (MB)
ASVBM	SVDSS	38154	14142	35858	2459	51.6	73.0	60.4	97.8	675.5	7313.2
	DeBreak	44908	**8886**	29104	**99**	60.7	**83.5**	70.3	95.5		
	Sniffles2	47362	12035	26650	1228	64.0	79.7	**71.0**	97.3		
	pbsv	45507	12275	28505	718	61.5	78.8	69.1	**98.3**		
	cuteSV	30261	11277	43751	1174	40.9	72.9	52.4	97.2		
	SVIM	**51510**	42845	**22502**	3230	**69.6**	54.6	61.2	98.1		
Truvari	SVDSS	37474	15478	36404	N/A	50.7	72.3	59.6	N/A	240.1	310.1
	DeBreak	45392	**8653**	28486	N/A	61.4	**83.1**	**70.7**	N/A		
	Sniffles2	46871	12736	27007	N/A	63.4	77.7	69.8	N/A		
	pbsv	38706	17545	35172	N/A	52.4	67.4	58.9	N/A		
	cuteSV	29742	12048	44136	N/A	40.3	69.7	51.1	N/A		
	SVIM	**50863**	44581	**23015**	N/A	**68.8**	52.1	59.3	N/A		
svbench	SVDSS	36503	14322	37448	N/A	49.4	71.8	58.5	N/A	812.9	617.2
	DeBreak	43688	**8938**	30263	N/A	59.1	**83.0**	**69.0**	N/A		
	Sniffles2	It could not be run in our experiments.							N/A		
	pbsv	42030	22024	31921	N/A	56.8	65.6	60.9	N/A		
	cuteSV	28915	25309	45036	N/A	39.1	53.3	45.1	N/A		
	SVIM	**49127**	131997	**24824**	N/A	**66.4**	27.1	38.5	N/A		

Note: The benchmark set contains a total of 74,012 variants. ASVBM and Truvari evaluated only on autosomes and sex chromosomes. ASVBM uses a relaxed matching mode, while Truvari employs the parameters --*dup-to-ins* and --*pick multi*; the benchmarking results of both are consistent. For svbench, the total time and peak memory are calculated based only on the datasets where it successfully executed, as it failed to run on some inputs. N/A: not applicable. Rc stands for Recall, Pr stands for Precision, and F1 stands for F1 score.

The SV matching criteria considerably impact the benchmarking results. Some callers might report duplications as insertions due to the functionalities and compatibility of their aligners. To account for this, ASVBM employs two matching parameters: strict matching, requiring exact SV type matches, and loose matching, allowing matches between insertions and duplications (Supplementary Note 2). Truvari also supports treating duplications as insertions, and we benchmarked both modes using a single user callset. Benchmarking results with different options for SV matching revealed the existence of duplications that satisfied five matching criteria. The trends observed in ASVBM results demonstrated a high degree of consistency with those obtained from the Truvari analysis. In the ASVBM results, under loose matching, pbsv exhibited approximately 20.8 % more TPs, indicating a tendency to identify insertions as duplications. Similar trends were observed in other callers, suggesting fewer matches for duplication variants under strict matching. Unlike insertions, duplications typically lack resolved sequences; therefore, these are not subject to sequence comparisons. In VCF files, SVs such as duplications are often represented using symbolic alleles (<DUP>), which describe the type of variant rather than providing the exact duplicated sequence. ASVBM handles this by omitting sequence comparison for unresolved duplications to avoid misleading similarity scores. However, when the sequence of a duplication is explicitly provided, it is fully incorporated into the similarity calculation, which is similar to insertions or deletions. Consequently, when comparing variants using tools such as Truvari, duplications represented as symbolic alleles may result in fewer matches due to the lack of precise sequence information, leading to a lower match count (Supplementary Table S1).

### 3.2 Performance improvement in local variant joint analysis validation

**Fig. 3:**
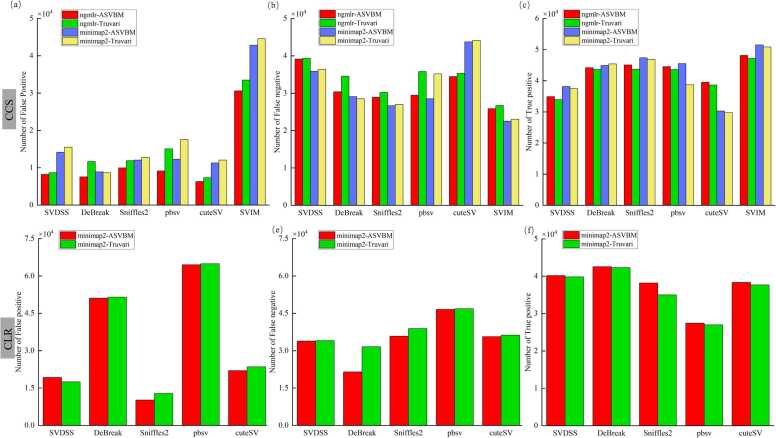
**Comparison of local variant joint analysis and traditional evaluation using the HG002 PacBio CCS and CLR data.** (a-c) Statistical results for HG002 PacBio CCS data aligned using ngmlr and minimap2. (d-f) Statistical results for HG002 PacBio CLR data aligned using minimap2.

To investigate the impact of local joint validation analysis benchmarking on the performance metrics of SV callers, we used several variant subsets from different aligners and evaluated six state-of-the-art variant callers mentioned previously using ASVBM. The results of SVDSS were excluded from the comparison of the CLR data, as it detected only 190 TPs, accounting for approximately 0.26 % of the benchmark set. In comparisons across different aligners, we also observed biases where several smaller variants were almost equivalent to larger variants. These alignment-induced SV size biases primarily stem from the mapping strategies employed by the aligners. For instance, ngmlr[49] divides reads into non-overlapping 256 bp sub-reads and maps them independently, in which minimap2 identifies high-similarity regions through a seed-extension algorithm. Compared to existing methods that match individual SVs, local joint validation analysis results in lower estimates of error rates (Fig. 3). The results of the joint analysis are closely related to the strategies employed by SV detection tools. For example, in the case of DeBreak—which does not rely on traditional alignment-based detection strategies, the improvement in the reported performance metrics under joint analysis is less pronounced. This observation suggests that SV detection methods employing traditional alignment strategies tend to exhibit greater increases in estimated performance when evaluated with joint analysis. This may be because local assembly-based approaches, such as DeBreak, have already reconstructed genomic regions de novo, thereby reducing the impact of alignment ambiguities that joint analysis is designed to mitigate. To further investigate the effect of sequencing modalities on the reported performance of SV callers, we analyzed CLR and CCS data. Fig. 3 illustrate significant differences in performance between the two sequencing modes. CLR data, with its higher error rate, particularly in repetitive or complex SV regions—limits the effectiveness of joint validation analysis and makes it harder to distinguish true variants from sequencing artifacts. In contrast, CCS data, with its lower error rate, provides more precise alignments and variant calls, enhances joint validation analysis and yields a greater improvement in SV detection accuracy. These findings highlight the critical role of data quality in SV benchmarking. Joint analysis reveals the actual performance of variant callers by identifying potential differences in variant representations between the callset and benchmark sets. Its effectiveness is inherently dependent on the underlying sequencing accuracy, with CCS data benefiting more than CLR.

To highlight the importance of incorporating the LP metric in the benchmarking of local joint variant analysis, we counted the number of LPs and TPs supported by merged variant calls (Supplementary Table S2). The results revealed that the number of LP reflects the capacity of the local joint analysis to provide a more comprehensive evaluation of SV caller performance. A substantial proportion of TPs was matched through the joint validation of two adjacent variants. Furthermore, we also observed instances where a single larger variant in the benchmark set required merging more than three consecutive variants from the callset for validation, with some instances involving the merging of six consecutive variants (Supplementary Figure S6 and Table S2). Traditional benchmarking methods, primarily comparing individual variants, typically misclassify consecutively identified smaller variants as FPs due to insufficient size similarity. This misclassification leads to an erroneous interpretation that the corresponding larger variants in the benchmark set are classified as FNs (Supplementary Figure S7), thereby limiting the precision and reliability of SV detection performance evaluation.

To further explore the relationship between LPs and TPs, we analyzed the number of variants contributing to each TP across different variant calling sets (Supplementary Table S2). Significant differences were observed in the benchmarking results across aligners, which can be attributed to the various strategies adopted by aligners for handling repetitive sequences, indel size thresholds, and error tolerance mechanisms. Additionally, the quality of alignment directly influences the sensitivity and accuracy of read alignment-based SV detection methods. Although these detection methods leverage read alignment, it is essential to note that categorizing a tool solely as alignment-based may not always be accurate in our analysis, as some tools integrate additional strategies or exhibit behaviors that extend beyond traditional alignment-based methods.

In contrast, read-based strategies are heavily influenced by aligners. For example, DeBreak can be considered a hybrid approach that combines read alignment and local assembly for SV detection. Among the benchmarking results, DeBreak reported the lowest number of LPs and validated only 49 TPs by merging. In contrast, tools such as pbsv and cuteSV have relatively fewer TPs than other methods. pbsv uses global alignment, signature extraction, and joint optimization to address errors introduced by local alignment-induced fragmentation. cuteSV, on the other hand, employs a heuristic approach to extract and merge large insertion/deletion features from the CIGAR string, aiming to reconstruct complete SV features fragmented into multiple small indels due to alignment.

### 3.3 Comprehensive benchmarking with ASVBM

The benchmarking results for multiple user callsets generated by ASVBM indicate that Sniffles2 outperformed other callers by 0.7 %-18.6 % on the F1 score (71.0 %) while achieving the second highest recall (64.0 %), demonstrating robust performance in SV detection (Supplementary Figure S8). SVIM called the largest number of SVs among all tools and achieved the highest recall (69.6 %); however, this came at the expense of lower precision (54.6 %). In addition, pbsv achieved a relatively high sequence similarity score (98.3 %), consistent with previous results[50]. To further refine the analysis, we stratified the performance metrics by SV size category. A significant proportion of variants (approximately 70 % of TPs) fell within the 1–100 bp range compared to the other seven categories (Supplementary Figure S9). Notably, SVIM exhibited relatively high recall performance across various size ranges. Overall, a decreasing trend in recall was observed as SV size increased, suggesting limitations in detecting larger variants (Supplementary Table S3).

The performance of various benchmarking tools, such as ASVBM, Truvari, critically depends on the representation of genomic regions. To demonstrate the interpretability of joint analysis, we evaluated the chr1:801,800–802,400 region of the LINC01128 gene. In this region, we observed a 147 bp insertion in the benchmark set. This insertion was split into two segments during alignment, resulting in apparent insertions of 49 and 98 bp. Manual inspection revealed that five out of six query VCFs correctly identified one or both of these variants, except for DeBreak. DeBreak, which relies on local consensus/assembly, detected a single 146 bp insertion. Other callers such as SVDSS, cuteSV, Sniffles2, and SVIM reported two records. Since the joint analysis validated both variants, they should not be considered FPs. ASVBM correctly accounted for all variants, as expected. However, Truvari, which compares SVs individually, labeled both calls as FPs. These differences in the local integrative analysis accounted for the higher number of TP variant calls reported in Table 2. ASVBM enables the benchmarking of multiple user callsets with a single command, generating mutual comparison results and creating an upset plot to statistically present the number of overlapping TPs (Supplementary Figure S10).

ASVBM analyzed the *breakpoint distance* between the results from different methods, which measures the deviation in breakpoints. A value closer to 0 indicates higher consistency in the reported SV positions, whereas a greater deviation from 0 suggests less precise breakpoint calling. Within the ± 50 bp range, SVDSS had the highest proportion of calls, followed by DeBreak and Sniffles2 (Supplementary Figure S11). ASVBM also computed the *variant size ratio* for overlapping variants across different identification results, which assesses the completeness of the identified SV regions. In some instances, there may be imprecise identification results with substantial discrepancies in length. Similar inconsistencies can arise when the same variant is split into adjacent items or when adjacent items are misidentified as a single variant. Among the evaluated results, SVIM called the highest number of SVs within the 0.7–1.2 range, indicating a relatively accurate length estimation for a large portion of its calls (Supplementary Figure S12). Furthermore, ASVBM facilitates a comparative analysis of results from various SV callers by simultaneously benchmarking multiple user callsets against a given reference set (Supplementary Note 3). Partial benchmarking results for multiple user callsets are presented in an HTML screenshot (Supplementary Figure S13).

### 3.4 Analysis of allele-matching statistical results

A major challenge in comparing SV calling tools is the inherent imperfection of available callsets, particularly in their ability to distinguish allelic variants. Within the 2020 GIAB v.0.6 callset Tier 1 regions, approximately 7.6 % of SVs occupied identical positions on the same chromosome, representing allelic SVs. The presence of these variants introduces additional complexity and poses challenges for SV calling, while also providing an opportunity to assess the performance of SV callers in handling complex genomic regions. To facilitate a more comprehensive analysis of the user callsets, multi-allelic variants were extracted from Tier 1 regions and used as a benchmark set. Subsequently, the user callsets specific to alleles were constructed by selecting variants that were proximal to the allelic SV regions based on reference distances determined by variant size. Then, resulting callsets were employed to evaluate the performance of SV callers in calling multi-allelic SVs, a particularly challenging task in current benchmarking efforts.

As anticipated, all detection methods achieved greater precision than the Tier 1 region. Detailed examples of allele matching are provided in Supplementary Figure S14, S15. Benchmarking against allelic SVs offered a more comprehensive assessment of state-of-the-art SV detection methods (Supplementary Table S4). The results of this experiment followed the same trend as the complete genome analysis, with SVIM achieving the best F1 score (72.7 %), outperforming other approaches. DeBreak reported the fewest FPs (145) among 3067 positive calls, resulting in the best precision (95.5 %). The different tools exhibited similarly high precision (ranging from 88.3 % for SVIM to 93.8 % for pbsv). Sniffles2, and cuteSV demonstrated comparably high recall and F1 scores. SVIM outperformed other callers in recall by 4.1 %-14.3 %, suggesting its stronger capability in identifying multi-allelic SVs. In conclusion, SVIM identified the most TPs (3475), exceeding other callers by 6.8 %-23.1 %. This trend displayed general similarity to the benchmarking results of Truvari, although some performance differences can be attributed to distinct approaches in allelic matching. ASVBM matches a query variant, regardless of its zygosity, to the corresponding baseline variants in the benchmark set. If the query and benchmark variants independently met the predefined matching criteria, they were classified as TPs. In contrast, Truvari matched a query homozygous variant to a heterozygous variant in the benchmark set and considered both to be TPs[51]. These differences in matching strategies may lead to variations in TP classification, particularly for allelic and complex SVs. Of course, no single method is absolutely optimal; instead, each method has strengths for specific applications. Users should select the appropriate tool based on their downstream objective.

## 4 Discussion

In this study, we introduce ASVBM, an improved benchmarking tool designed to address challenges by incorporating the concept of latent positives and adopting a strategy of local joint validation analysis. The performance improvements stem from recognizing that multiple smaller variants in the callset typically correspond to a larger variant in the benchmark set, effectively overcoming key limitations of traditional benchmarking approaches. We demonstrate the superior performance of ASVBM in reducing false positives and false negatives, particularly under conditions of alignment inaccuracies. By accounting for latent positives, ASVBM resolves ambiguities arising from differences in alignment strategies, thereby improving benchmark objectivity and providing a more comprehensive benchmark for SV detection methods. The stratification of performance metrics by SV size offers detailed insights into the detection capabilities of various methods, revealing size-related trends in precision and recall that are critical for method optimization. A notable advantage of ASVBM lies in its ability to benchmark multiple user callsets simultaneously with a single command, generating detailed benchmarking reports.

SV benchmarking aims to assess the concordance between called variants and a reference benchmark set. Given the inherently high error rates of long-read sequencing data and the fragmentation of CIGAR signatures caused by inaccuracies or insufficiencies in read alignment, different SV detection pipelines may produce diverse representations of the same SV event, posing challenges for read-alignment-based detection methods. We demonstrate the ability of ASVBM to benchmark the identification results of six SV callers using five criteria that incorporate novel concepts such as latent positives and an improved sequence similarity metric, and compared it with three other benchmarking tools. According to the local joint validation analysis strategy, ASVBM identifies the equivalence between multiple smaller variants in the callset and larger variants in the benchmark set, thereby mitigating mismatches arising from differences in variant representation rather than true calling errors, and enabling a more objective and equitable evaluation of SV calling performance. Current benchmarking tools, including ASVBM, may facilitate matching between duplications and insertions. Setting sequence similarity to 0 is recommended when using this option in Truvari, which may impact the similarity assessment for insertions and deletions (Supplementary Note 4). However, when considering sequence similarity, it is equally important to ensure the proper handling of comparisons between duplications and insertions, as well as among other SV types. Therefore, a more refined and accurate approach is essential for handling these cases to improve the reliability of SV matching.

In this study, we offer a versatile approach to benchmark user callsets against a reference benchmark set. This flexibility extends across sequencing platforms, as ASVBM relies solely on user call results and their corresponding reference sequences. Alternative methods such as TT-Mars[52] assess SV calls based on haplotype-resolved assemblies without requiring a predefined truth set. However, these approaches tend to be more computationally intensive and lack direct false-negative assessment. In contrast, ASVBM provides a streamlined and efficient framework for comprehensive SV caller benchmarking when a high-quality benchmark set is available. However, it should be noted that ASVBM is currently more effective for resolving SVs (i.e., deletions, insertions, inversions, and duplications). The accuracy of SV matching is related to the study design. User callsets may contain SVs with imprecise breakpoints or insufficient sequence resolution, which may impact the matching process. Further development is necessary to comprehensively address the challenges associated with complex genomic rearrangements[53] or pipelines that generate highly divergent SV representations.

Besides, the benchmarking results were stratified by variant size, with detailed results reported for each size range, providing valuable insights for improving SV detection algorithms. As the size of the SVs increased, all methods exhibited a decreasing trend in recall, highlighting the potential for performance improvements in larger SV regions. The benchmarking results provide a detailed breakdown of SV events identified by various SV detection methods, enabling further analysis and comparison. The records in the results can be checked, analyzed, and visualized using the Integrative Genomics Viewer [54] (IGV). By focusing on read alignment depth, breakpoint support, and assembly concordance in repetitive regions, developers could potentially address observed discordant calls to improve the accuracy of SV detection algorithms. With advances in sequencing technologies and improvements in SV detection methods, the proportion of concordant SVs is expected to increase further.

Although ASVBM currently addresses some key aspects of SV benchmarking, it has some limitations worth mentioning. Firstly, our work is effective for calculating sequence similarity only in insertions and deletions where sequence-resolved calls are present, and does not consider sequence similarity for duplications, inversions, and translocations, which generally lack sequence-resolved calls. Furthermore, missing variants and potential mispredictions affect nearly all published callsets, even those of the highest quality, which have been reported to have approximately 5 % false discovery rate (FDR) and a much higher false negative rate [37]. Therefore, the 2020 GIAB v0.6 callset used in our experiments may have certain limitations. One potential area for improvement involves addressing the issue of shared FNs (reported in common across multiple user callsets compared to the benchmark set) and incorporating shared FPs (variants classified as FP in multiple user callsets) into the benchmark set, which could enhance the robustness and reliability of the benchmarking process. Building on these considerations, potential refinements could involve expanding SV matching criteria, exploring diverse graphical styles to enhance visualization, and improving the overall clarity and presentation of generated images.

## CRediT authorship contribution statement

**Chaoqun Zhu:** Investigation. **Fei Wang:** Methodology. **Wei Quan:** Conceptualization. **Yuanjun Ma:** Formal analysis. **Yucui Dong:** Writing – review & editing, Supervision, Methodology, Conceptualization. **Xiao Zhu:** Writing – review & editing, Software, Project administration, Methodology, Conceptualization. **Peizheng Mu:** Writing – original draft, Validation, Software, Methodology. **Xiangyan Feng:** Writing – review & editing, Validation, Formal analysis. **Lanxin Tong:** Investigation, Formal analysis. **Jie Huang:** Validation.

## Funding

This work was supported by a grant from the 10.13039/501100001809National Natural Science Foundation of China (61902094, 62102346 and 81402054), the Shandong Provincial Natural Science Foundation of China (ZR2021MH036 and ZR2021QF123), the Science and Technology Program of Binzhou Medical University (50012304325), and the Doctoral Scientific Research Foundation of Yantai University (JS22B171 and JS22B16).

## Data and code availability

The HG002 PacBio CCS data and PacBio CLR data were downloaded from the National Center for Biotechnology Information (NCBI) with accession numbers SRX5327410 and SRX7668835. The 2020 GIAB v.0.6 callset Tier 1 benchmark set for reference genome GRCh37 was downloaded from the GIAB (ftp://ftp-trace.ncbi.nlm.nih.gov/giab/ftp/data/AshkenazimTrio/analysis/NIST_SVs_Integration_v0.6). The human reference genome GRCh37 was downloaded from the IGSR FTP server at the following link: ftp://ftp.1000genomes.ebi.ac.uk/vol1/ftp/technical/reference/phase2_reference_assembly_sequence/hs37d5.fa.gz. All tests were performed on a Linux x86_64 workstation equipped with an Intel(R) Core (TM) i9–10980XE CPU @ 3.00 GHz; the system featured 36 logical processors, and 25344 KB of shared L3 cache. The memory allocation was 200 GB unless otherwise stated, and the number of CPU cores allocated was all unless otherwise noted. All source code used to analyze results is available at https://github.com/zhuxiao/asvbm. More detailed experimental information is available at https://github.com/zhuxiao/asvbm-experiments.

## Declaration of Competing Interest

The manuscript has been reviewed and approved by all authors listed. The authors declare that they have no conflicts of interest.
