# Supplementary material for: ASVBM: Structural variant benchmarking with local joint analysis for multiple callsets
Source: Comput Struct Biotechnol J. 2025 Jun 29;27:2851–62. doi: 10.1016/j.csbj.2025.06.045 (PMC12271604; doi:10.1016/j.csbj.2025.06.045)
Supplement: Supplementary file 1 — Supplementary material [file mmc1.docx]

**Supplementary Materials for ASVBM: Structural Variant Benchmarking with Local Joint Analysis for Multiple Callsets**

This file includes:
Supplementary Notes 1-4 (Pages 2-3)

Supplementary Figures S1-15 (Pages 4-21)

Supplementary Tables S1-4 (Pages 22-26)

1. **Supplementary Notes**

**Supplementary Note 1. Variant** **information extraction with ASVBM**

Prior to benchmarking, ASVBM extracts variant information from both the user callset and the benchmark set. This information includes essential elements for benchmarking: chromosome name (chrname), start position (startPos), end position (endPos), SV type (SVType), SV length (SVLen), reference sequence (REF), and variant sequence (ALT). However, some identified SV results may lack fields such as endPos, SVType, or SVLen. ASVBM leverages the REF and ALT fields within the user callset and benchmark set to analyze SV type and size. Additionally, when encountering multi-allelic variants at a single site (e.g., with tools like SVDSS), ASVBM may separate the consolidated variant information into distinct records. This ensures accurate comparisons and reliability benchmarking by maintaining consistent variant information across SVs. For unbalanced variants, ASVBM infers the type according to base count differences: deletions when REF bases exceed ALT bases, and insertions/duplications when ALT bases exceed REF bases. The formula for calculating the end position of precise variants is provided below. The formula for imprecise variants is identical, representing the best estimate:

$$endPos=startPos+length of REF allele-1$$

Translocation (TRA) typically involve breakpoints located either on different chromosomes or on the same chromosome. An arbitrary rearrangement event can be summarized as a set of novel adjacencies. Each adjacency ties together 2 breakends (BND). The two breakends at either end of a novel adjacency are called mates. For ASVBM, the information of two breakends of the mates is saved as one record. Some SV callers, such as SVIM and pbsv, may generate duplicate mate records in their output, while others do not. These duplicate mate records can be filtered out based on experimental requirements.

**Supplementary Note 2. Comparison to Truvari under different matching criteria**

SV matching criteria significantly impact the benchmarking results. For different variant sizes, appropriate reference distances are used for breakpoint matching. Specifically, a 200 bp search space extension is applied for variants under 100 bp, while a 1 kbp extension is used for larger variants. Some callers might report duplications as insertions due to their functionalities and compatible aligners. To account for this, ASVBM employs loose matching by default, allowing for matches between these variant types. Truvari, another SV evaluation method reporting precision, recall, and F1 score, offers similar insights through its bench command. Truvari's criteria were developed as part of the Genome in a Bottle consortium (GIAB) and are widely applicable for single-callset comparisons of a replicate to a ground-truth set of SVs. Therefore, we benchmarked the results of different SV callers on the same dataset both ASVBM and Truvari. Notably, the trends observed in ASVBM's results demonstrated a high degree of consistency with those obtained from the Truvari analysis.

**Supplementary Note 3. Experiment with multiple user callsets benchmarking**

This experiment utilized the following parameters: -*T* "*SVDSS;DeBreak;Sniffles2;pbsv;cuteSV;SVIM*" -*m* *50000* -*C* "*1;2;3;4;5;6;7;8;9;10;11;12;13;14;15;16;17;18;19;20;21;22;X;Y*", representing the names of SV callers, the maximal SV length, and set of chromosomes to be included in benchmarking. ASVBM then sequentially benchmarked each input datasets, collecting results (TP, FP, FN, LP, precision, recall, F1 score, and sequence similarity) for statistical charts. While capable of benchmarking various SV types (insertions, deletions, inversions, duplications, and translocations), this study focused on insertions and deletions in autosomes and the sex chromosomes (X and Y). To comprehensively assess caller performance and accuracy, ASVBM reported two categories of metrics: basic statistics (TP, FP, FN, LP) and performance metrics (recall, precision, F1 score and sequence similarity).

**Supplementary Note 4. Impact of loose matching on sequence similarity**

Due to alignment ambiguities, some SV callers may classify duplications as insertions. While treating duplications as insertions in benchmarking is useful, it introduces challenges for sequence similarity calculations, especially since duplications are often not sequence-resolved. Truvari (v4.0.0), a widely used benchmarking tool, recommends setting --*pctseq* 0 to disable sequence comparison for this reason. However, ignoring sequence similarity can lead to incorrect matches between variants with substantial sequence differences. For example, in the DeBreak benchmarking using the HG002 CCS dataset, a 1454 bp insertion on chromosome 1 (breakpoint at position 7580260) was classified by ASVBM as a false negative due to a low sequence similarity of 0.4934, whereas Truvari labeled it a true positive by ignoring sequence content. On the other hand, when sequence similarity is considered, limitations arise because duplications are often represented by symbolic alleles (e.g., <DUP>), which lack resolved sequence information and complicate similarity calculations. ASVBM supports sequence similarity comparisons for duplications that are sequence-resolved. For duplications without resolved sequences, ASVBM skips the sequence similarity step; variants of this type are considered matches if they satisfy other criteria such as SV type, reference distance, overlap, and size similarity.

1. **Supplementary Figures**

**
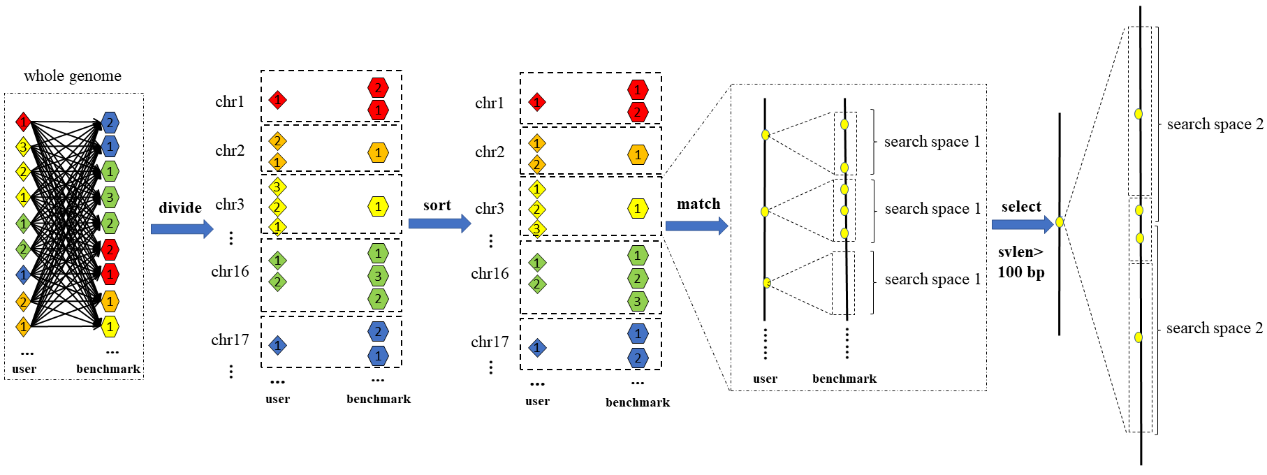
**

**Supplementary Figure S1. The matching calculation method between the SV calling result set and the benchmark set.** The specific steps are as follows: (1) divide the user callset and the benchmark set according to chromosomes to form multiple subsets; (2) sort the records in each subset according to the SVs positions; (3) Extend each data record of the SV identification result by 200 bp on both sides. If there are data records in the search space that satisfy the conditions of the same SV type and sequence similarity, they are considered as matched records; (4) for SVs larger than 100 bp, expand the search space of SV positions by 1 kbp on both sides, if a matching record with the same SV type and sequence similarity is found, consider them as matched SVs.


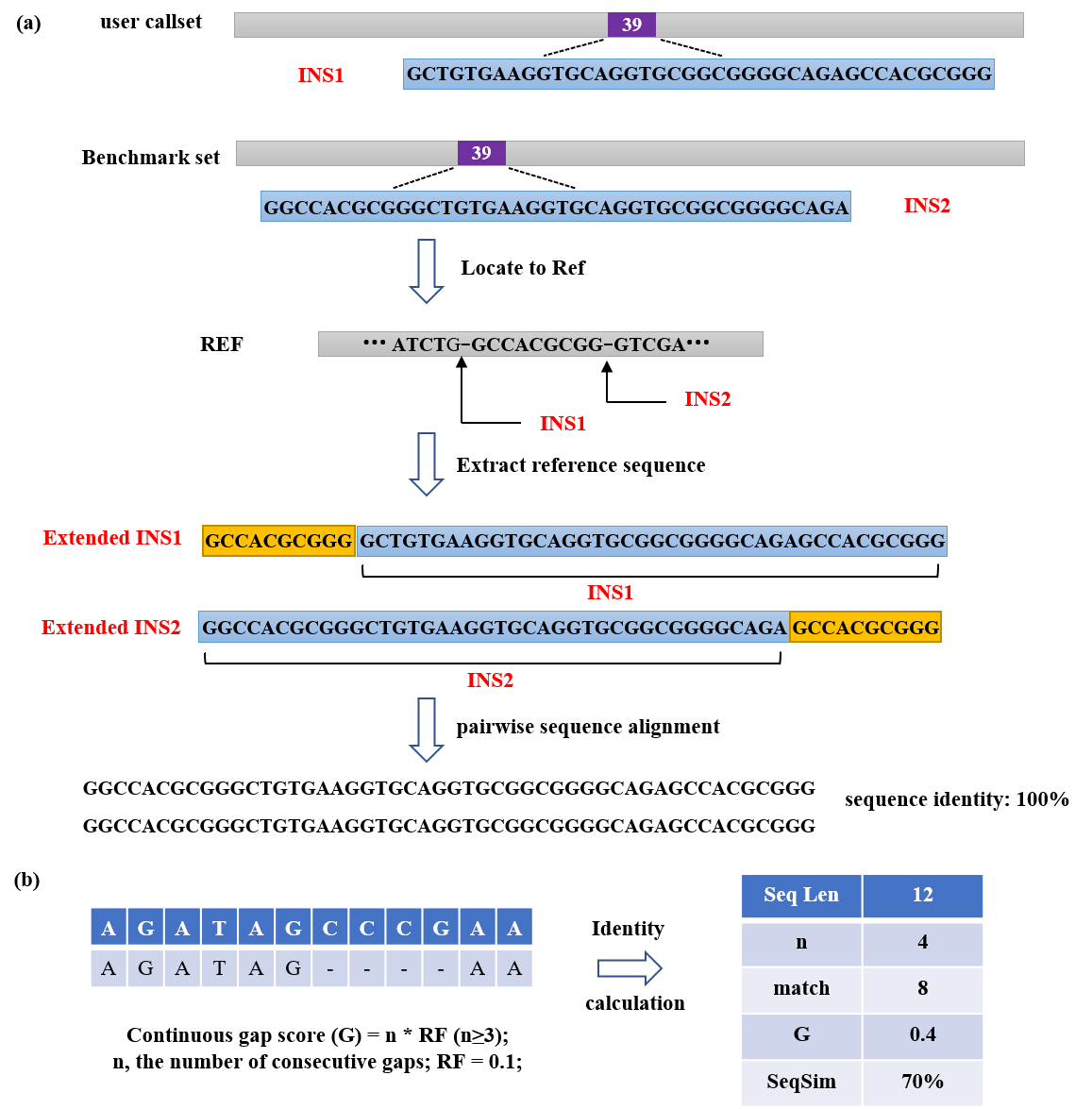


**Supplementary Figure S2. The sequence similarity calculation process between the SV calling result set and the benchmark set.** (1) Extract the corresponding variant sequences to be compared from the user callset and the benchmark set; (2) the reference sequence between the upstream-most start and downstream-most endpoints of the two SVs with different variant locations is extracted ; (3) Perform pairwise sequence alignment on the two variants after sequence extended in the reference sequences to find the optimal matching sequence; (4) Calculate sequence similarity for the generated optimal matching sequences and introduce relief factors to reduce the impact of consecutive gaps.


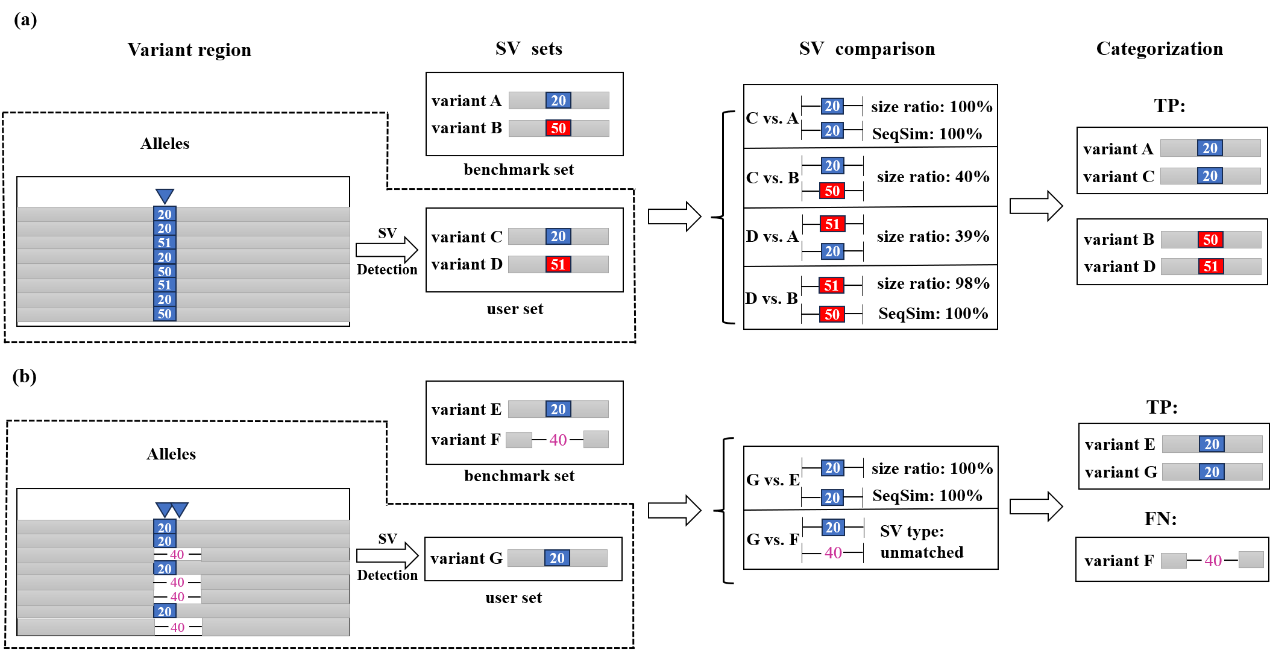


**Supplementary Figure S3. Workflow of allele matching using ASVBM.** Allelic SVs matching requires comparing each detected variant with its corresponding items in the benchmark set (2020 GIAB v0.6 callset). (a) Illustrates the matching process for allelic variants of the same SV type. (b) Demonstrates the matching process for allelic variants of different SV types. Matching is done according to criteria such as reciprocal overlap, SV type matching, size similarity, and sequence similarity. SVs satisfying these criteria are categorized as true positives, while others will be classified as false positives or false negatives.


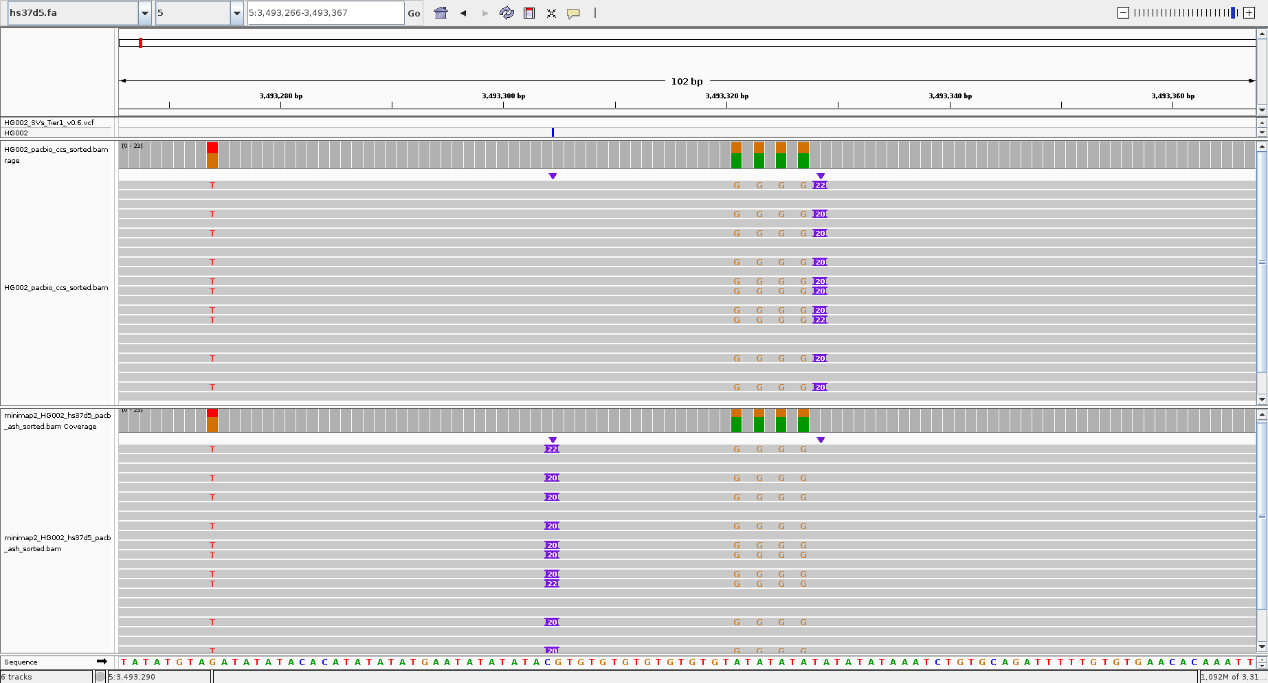


**Supplementary Figure S4. Examples of sequence similarity calculation in the presence of alleles and SNVs within SV regions.** In the IGV screenshot, the blue marker in the first track represents a variant in the benchmark set. The second track shows the alignment using NGMLR (used for SV calling), and the third track shows the alignment with minimap2. Due to differences in SV sites, extracting the reference genome sequence surrounding the variant becomes necessary. However, reads containing variants may also include SNVs that are inconsistent with the reference genome, which may potentially reduce the sequence similarity after extracting the reference.


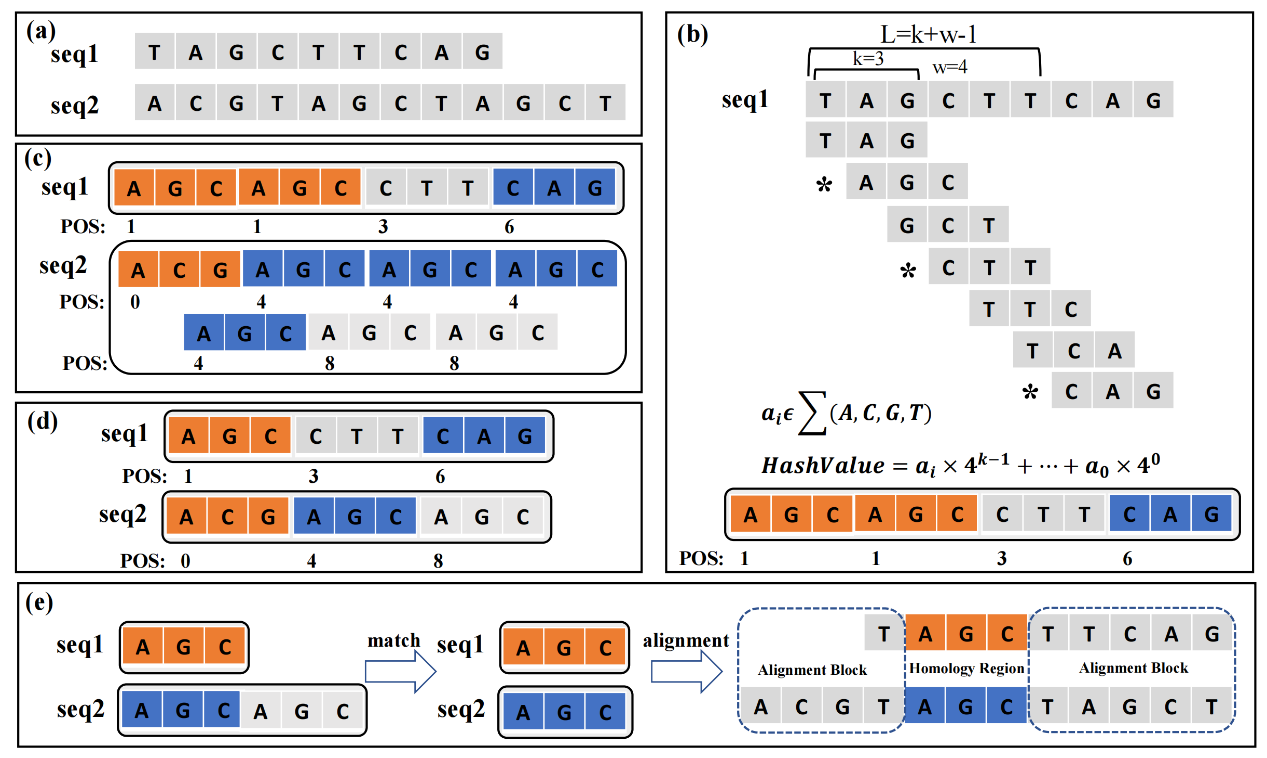


**Supplementary Figure S5. Examples of sequence similarity calculation with larger SVs by ASVBM.** (a) Extract the sequences for which sequence similarity variant needs to be calculated. (b) Compute the minimizer for each window of the sequence, which is the *k*-mers with the smallest hash value. In this example, we select k=3 and w=4. (b) shows that a total of 7 *k*-mers, with those marked by an asterisk (*) being the minimizer for each window. (c) Calculate the set of minimizers for each of the two sequences according to the method in (b). (d) Remove redundant items with the same locations from the minimizer sets. (e) Match items with the same hash value in the two minimizer sets according to proximity in location, and align the unmatching parts of the sequences (i.e., the regions within the dashed boxes), while the homology regions do not need alignment.


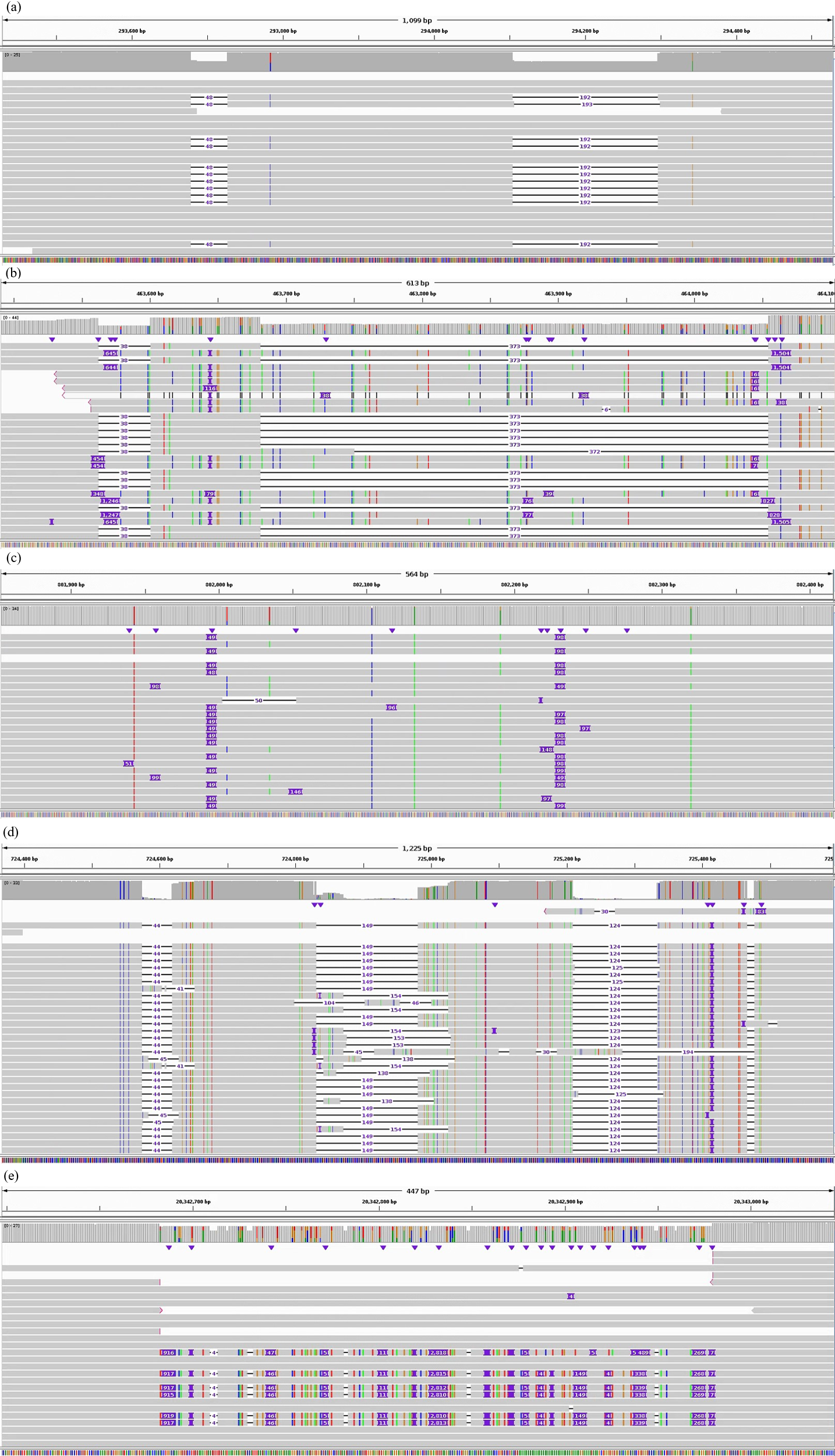


**Supplementary Figure S6. An example IGV schematic for validating local variants merging** **using HG002 PacBio CCS data.** (a), (b), and (c) are examples where two adjacent variants are merged and validated, resulting in a match that is entirely equivalent to a larger variant in the benchmark set. (d) shows a case where three adjacent variants are merged and validated, resulting in a record labeled as LP. The benchmark set reports a 311 bp deletion, but the detection results from different SV callers vary. SVDSS detects deletions of 44 bp, 149 bp, and 139 bp, which align well with the reported variation in the benchmark set. pbsv detects deletions of 44 bp and 154 bp. DeBreak, using a local assembly strategy, detects a 316 bp deletion. Sniffles2 detects deletions of 44 bp and 124 bp, while cuteSV only detects a 149 bp deletion. SVIM detects deletions of 44 bp and 147 bp. Among these, SVDSS confirms true positives matching the reference set through local variant joint analysis, while traditional benchmark tests classify them as false positives (FPs). Other callers, which fail to detect all variants in the region, cannot be validated through joint analysis and are also classified as FPs. In (e), the benchmark set contains an insertion of length 4564, which is matched to six smaller variants. The merged variant's length is highly similar to that of the benchmark variant. In traditional benchmarking, all six variants are classified as FPs, while the corresponding benchmark variant is labeled as FN. After merging and validation, the six variants are reclassified as LPs, and the benchmark variant is reclassified as TP.


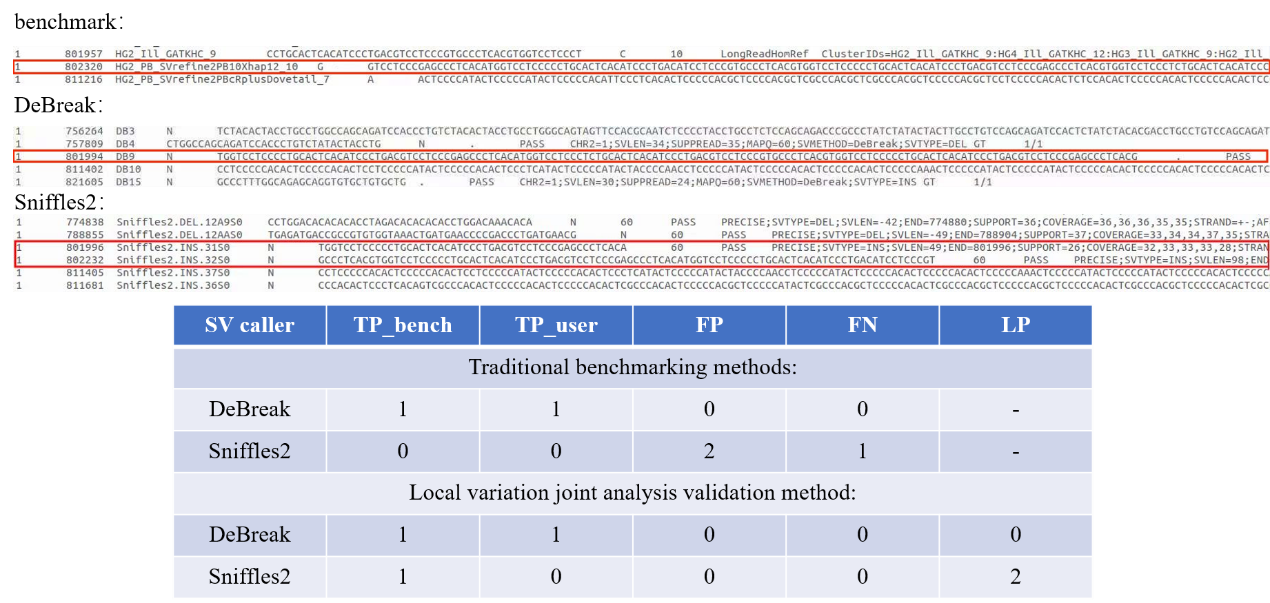


**Supplementary Figure S7. An example of benchmarking results from traditional benchmark and local variation analysis method using HG002 PacBio CCS data.** The use of local variation joint analysis for Sniffles2 detection results better addresses the comparison between multiple smaller variations in the user call set and larger variants in the benchmark set when merged.


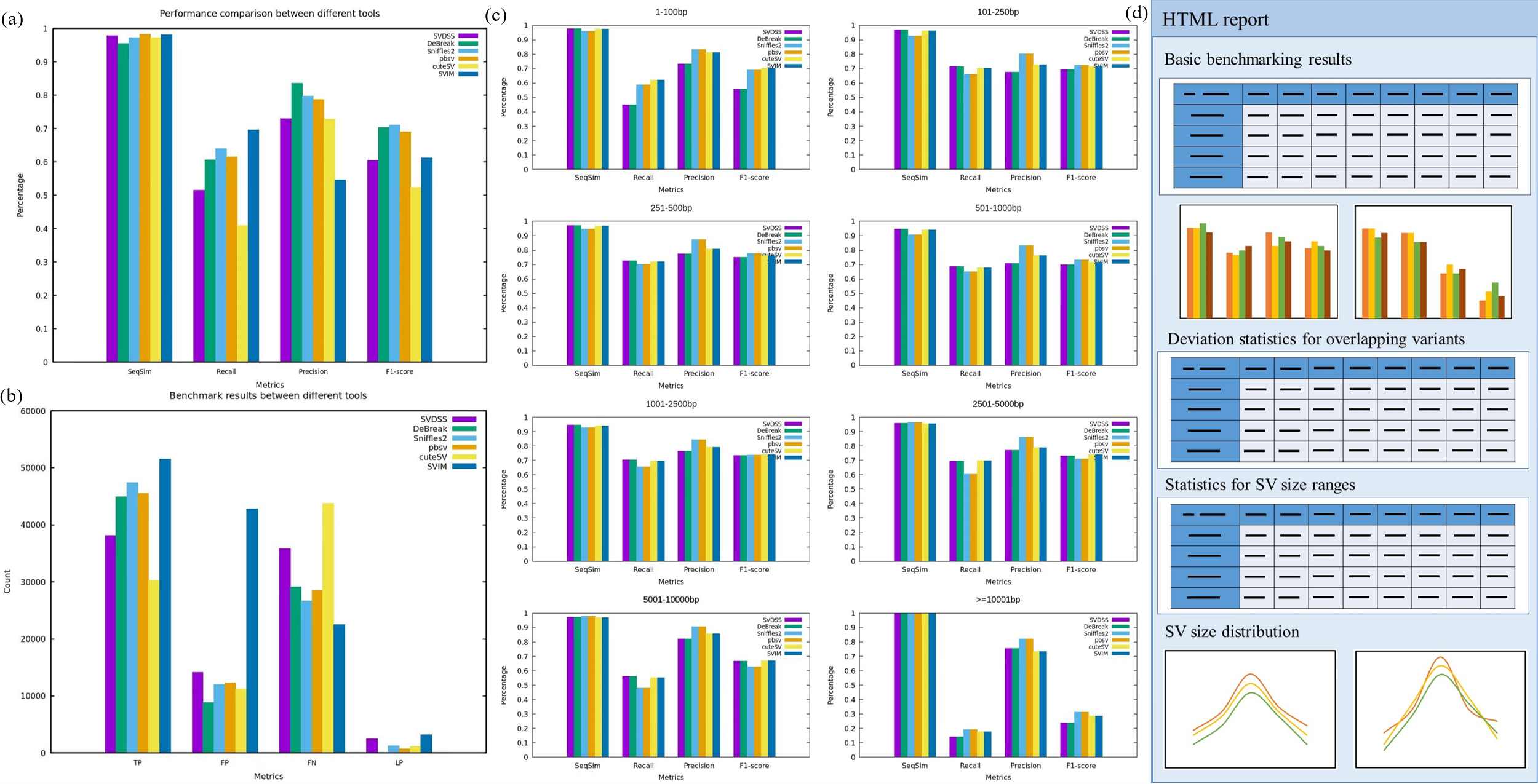


**Supplementary Figure S8. Statistics of SV identification results of different SV detection methods on autosomal and sex chromosomes X, and Y using the HG002 PacBio CCS data.** (a) Statistical results of basic metrics, including recall, precision, F1 score and sequence similarity (SeqSim), where sequence similarity refers to the sequence similarity of the SV that is matched and contained within the sequence. (b) Basic metric statistics, including TP, FP, FN and LP. (c) Statistics of SV identification results of different SV detection methods in different SV regions. (d) Cartoon of ASVBM stratification HTML report.


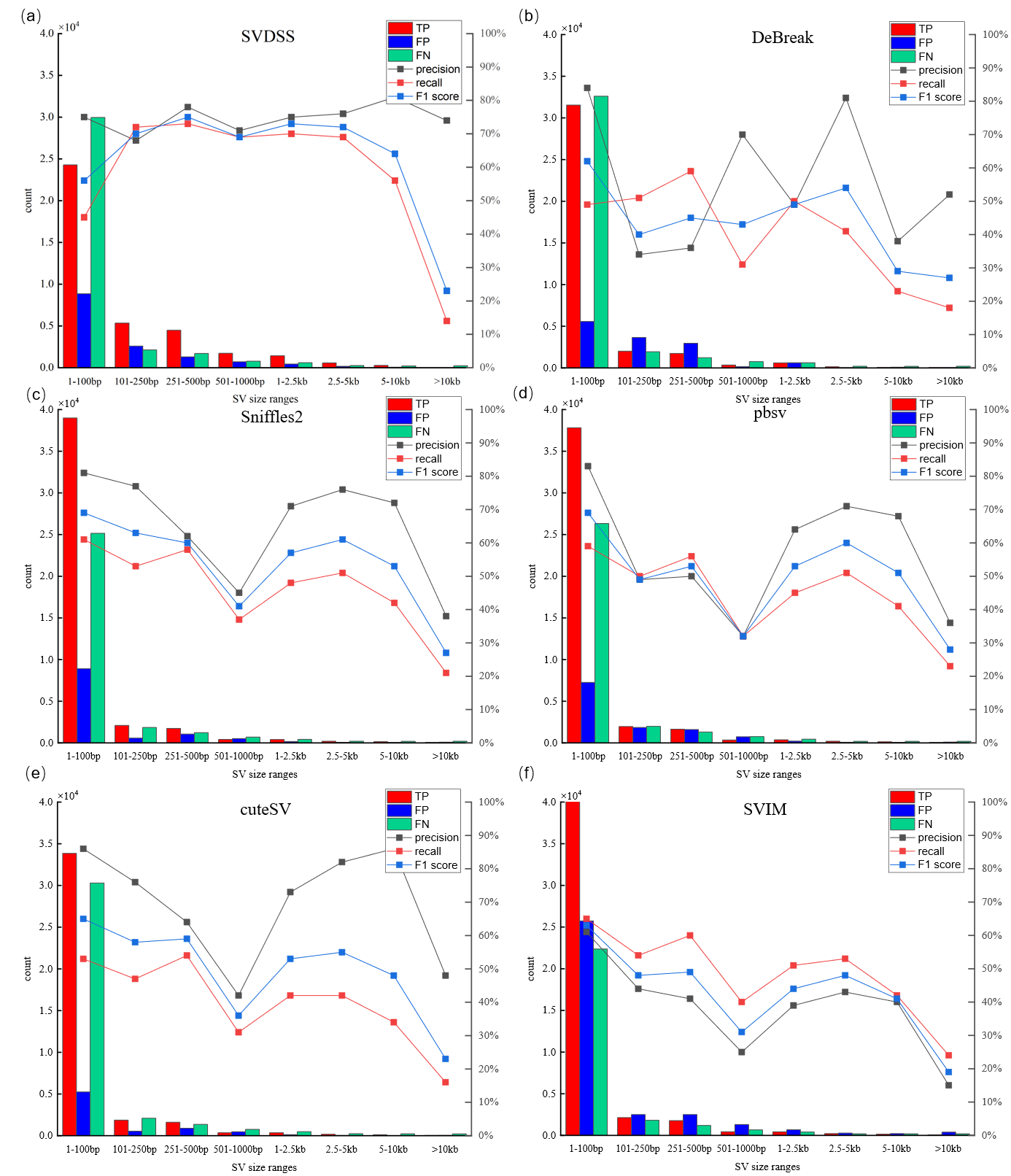


**Supplementary Figure S9.** **Statistics of different SV identification results with different SV size ranges using HG002 PacBio CCS data.** Results are shown that a significant portion of variants fall within the range of 1-100 bp, compared to other size categories. Nonetheless, different methods display varying detection capabilities across different sizes of SV regions, and overall, there is a decrease on the recall with the increase of SV sizes.


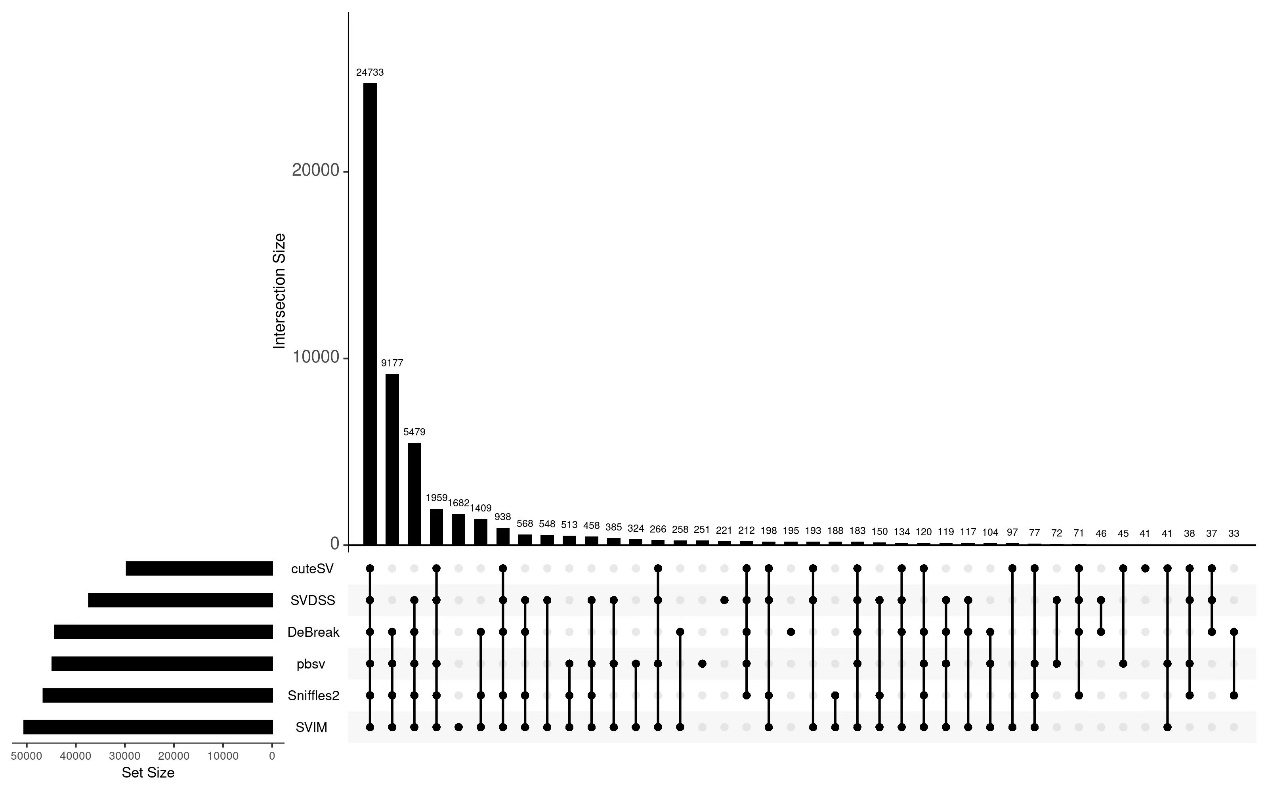


**Supplementary Figure S10. The upset plot of true positives records of multiple user callsets using HG002 PacBio CCS data.** Upset plot for TP records drawn from multiple user callsets. The total TP records of seven SV detection methods was 25486 records; SVDSS had the lowest number of unique TP records, followed by DeBreak.


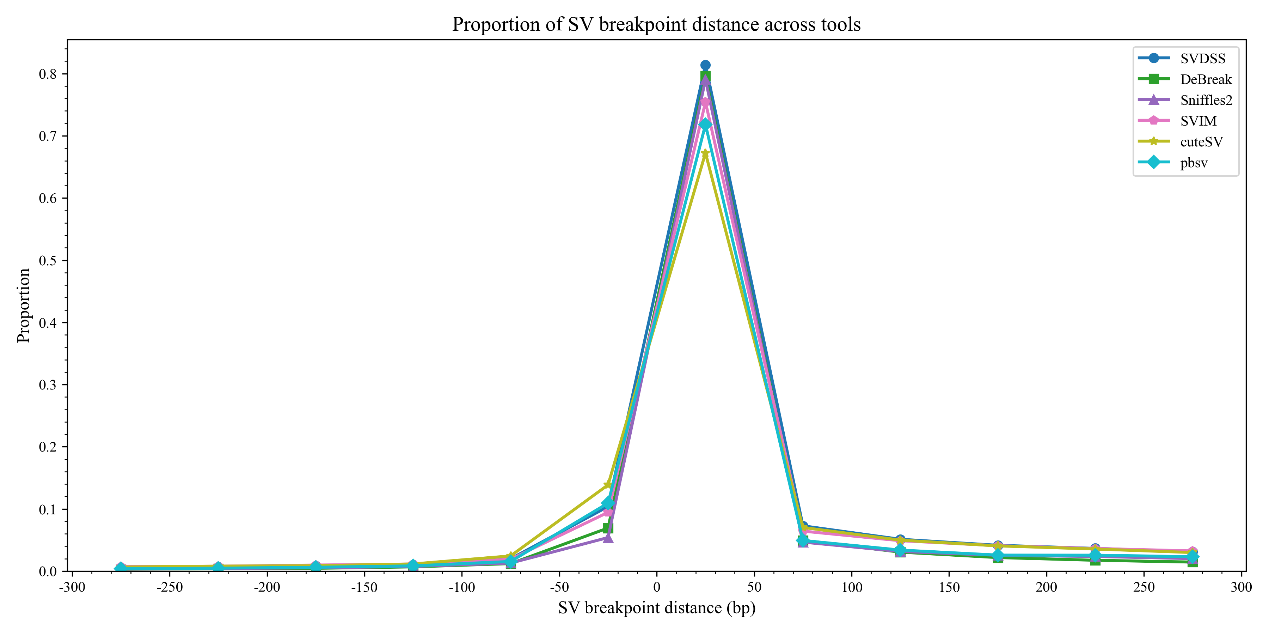


**Supplementary Figure S11. Comparison of the breakpoint distance of the SV results of different SV callers using HG002 PacBio CCS data.** For the breakpoint distance, the closer to 0, the more accurate the SV result. Among the six SV callers, SVDSS identified the highest proportion of SVs when the breakpoint distance of the range of 0-50 bp, and followed by DeBreak.


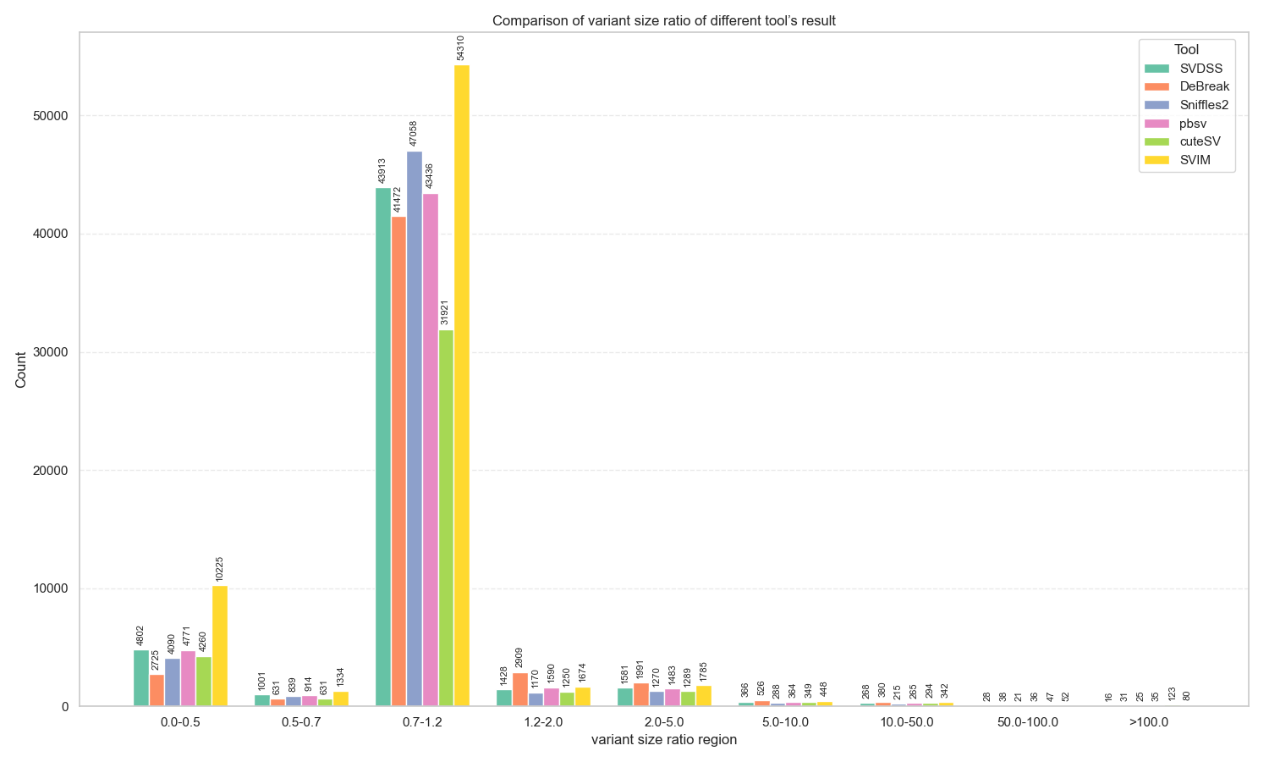


**Supplementary Figure S12. Comparison of variant size ratio of identification results of different SV identification methods using HG002 PacBio CCS data.** For the variant size ratio, the closer to the range of 0.7-1.2, the more accurate the SV identification result. SVIM had the largest proportion of SV identified in the range of 0.7-1.2, followed by Sniffles2 and SVDSS, and cuteSV had the smallest proportion.


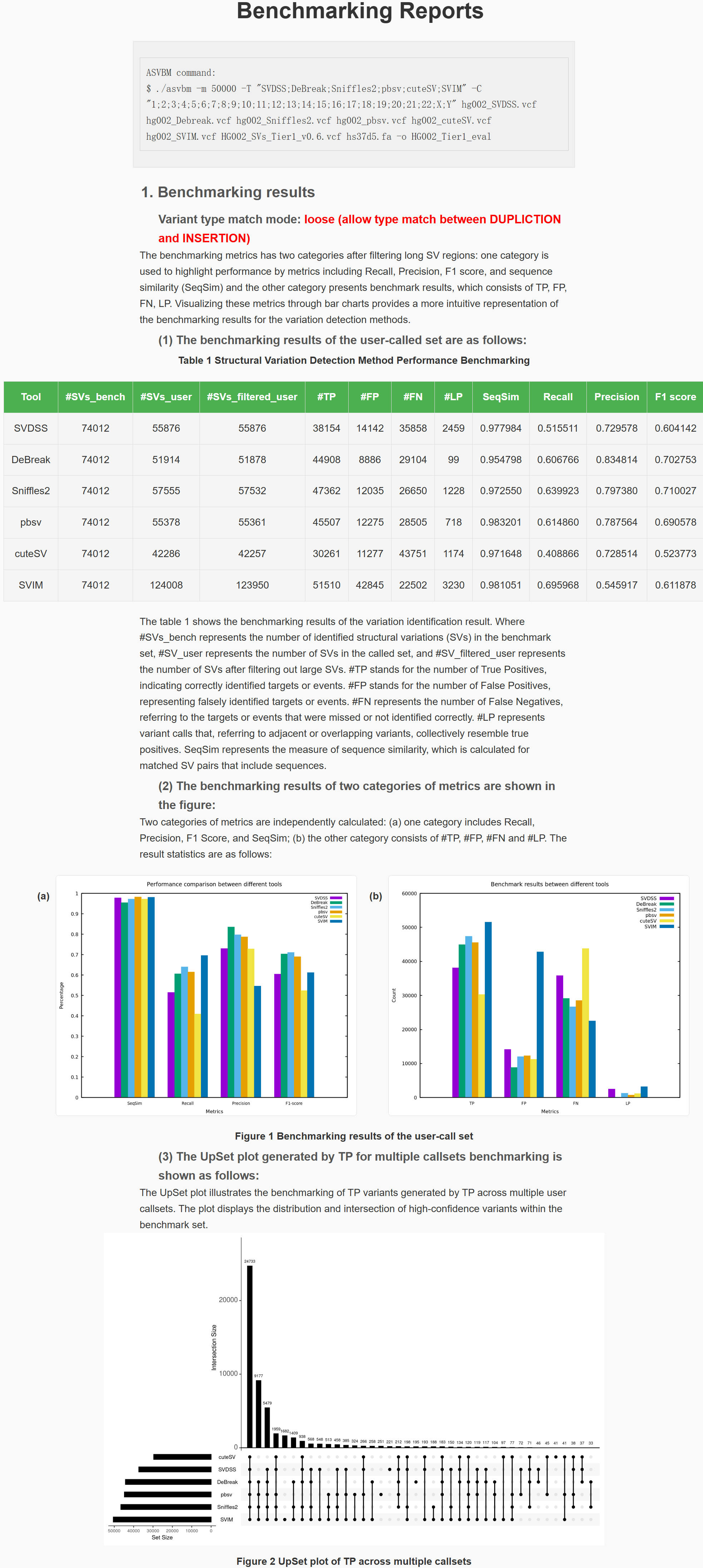


**Supplementary Figure S13. The HTML screenshot of the benchmarking results for multiple user callsets.** The HTML screenshot showed the benchmark command, mode for SV type matching, and calculation results for basic metrics, and also included statistical deviations for breakpoint distance and variant size ratio, as well as statistical results for basic metrics across various size ranges.


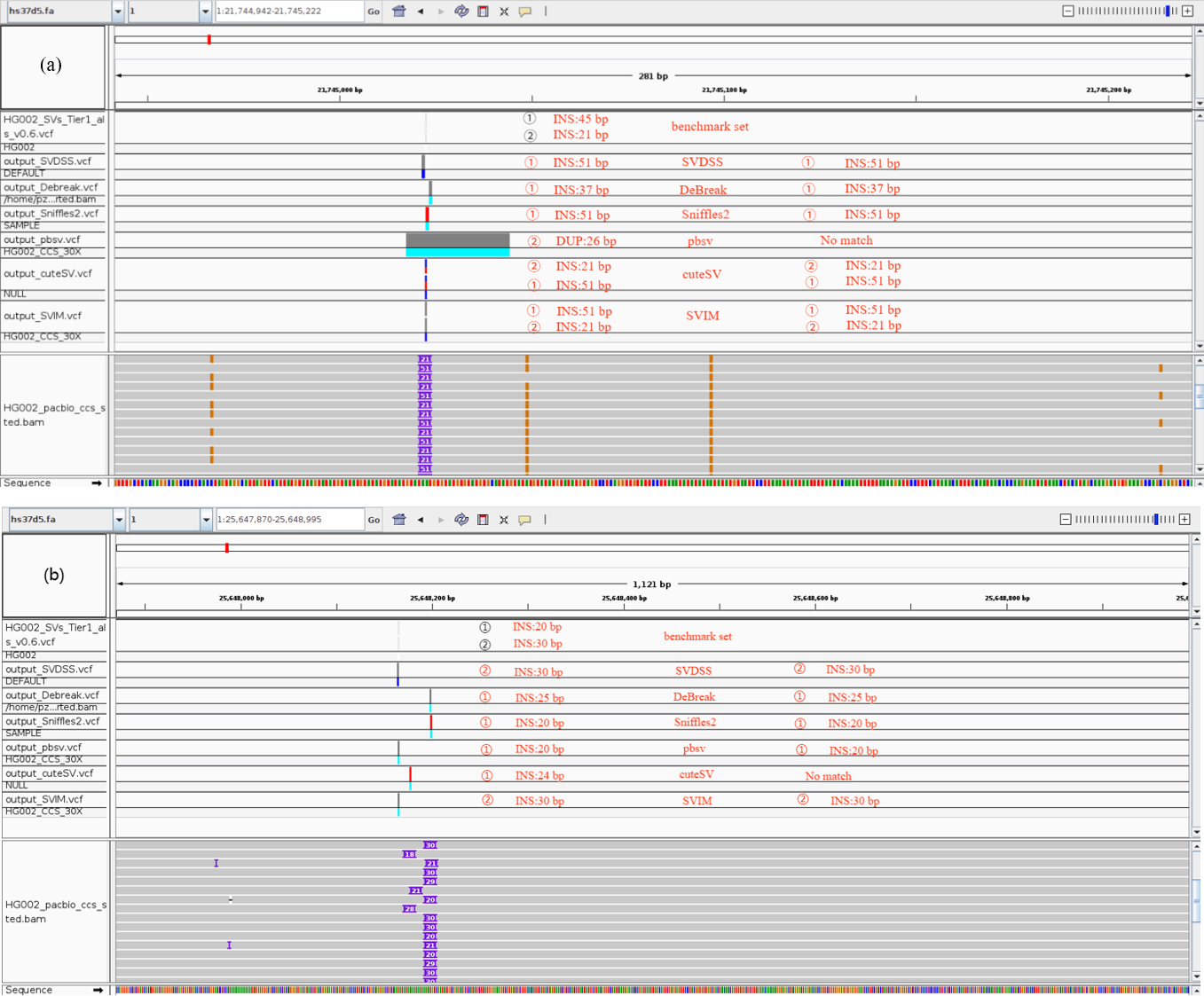


**Supplementary Figure S14. Examples of** **insertion SV regions with alleles have been accurately matched by ASVBM.** Within the benchmark set, there are two insertions of varying lengths, only one or two variants are identified in the detection results. The detection results of different methods are shown, and a comparison of the two variants could be made by matching the corresponding numbers within the red circles. On the left are the ASVBM matching results, and on the right are the Truvari matching results.

In Figure S14 (a), the variants are located at chr1:21745022-21745022 in the benchmark set, including two insertions of lengths 45 bp and 21 bp, respectively. It can be observed that the detection results vary among different methods. Specifically, cuteSV, and SVIM detect two different insertion variants, while other methods only detect one variant. For variant matching, SVs in the benchmark set that satisfy the matching criteria are considered as true positives (TP), such as those satisfying overlap, size similarity, sequence similarity, and SV type matching. SVDSS and Sniffles2 only match the variant with a length of 45 bp in the benchmark set, while the detection result of pbsv is a duplication (DUP) with a length of 26 bp, which will be considered as a TP if treated as an insertion. On the right, Truvari's results are consistent with ASVBM's results. In Figure S14 (b), at the locus chr1:25648165-25648165 in the benchmark set, allelic variants are observed, with lengths of 20 bp and 30 bp, respectively. This SV region, all tools identifying only one of the variants. On the right, SVDSS, Sniffles2, pbsv, and SVIM accurately identified one of the variants and provided precise matches, while DeBreak and cuteSV yielded less accurate detection results.


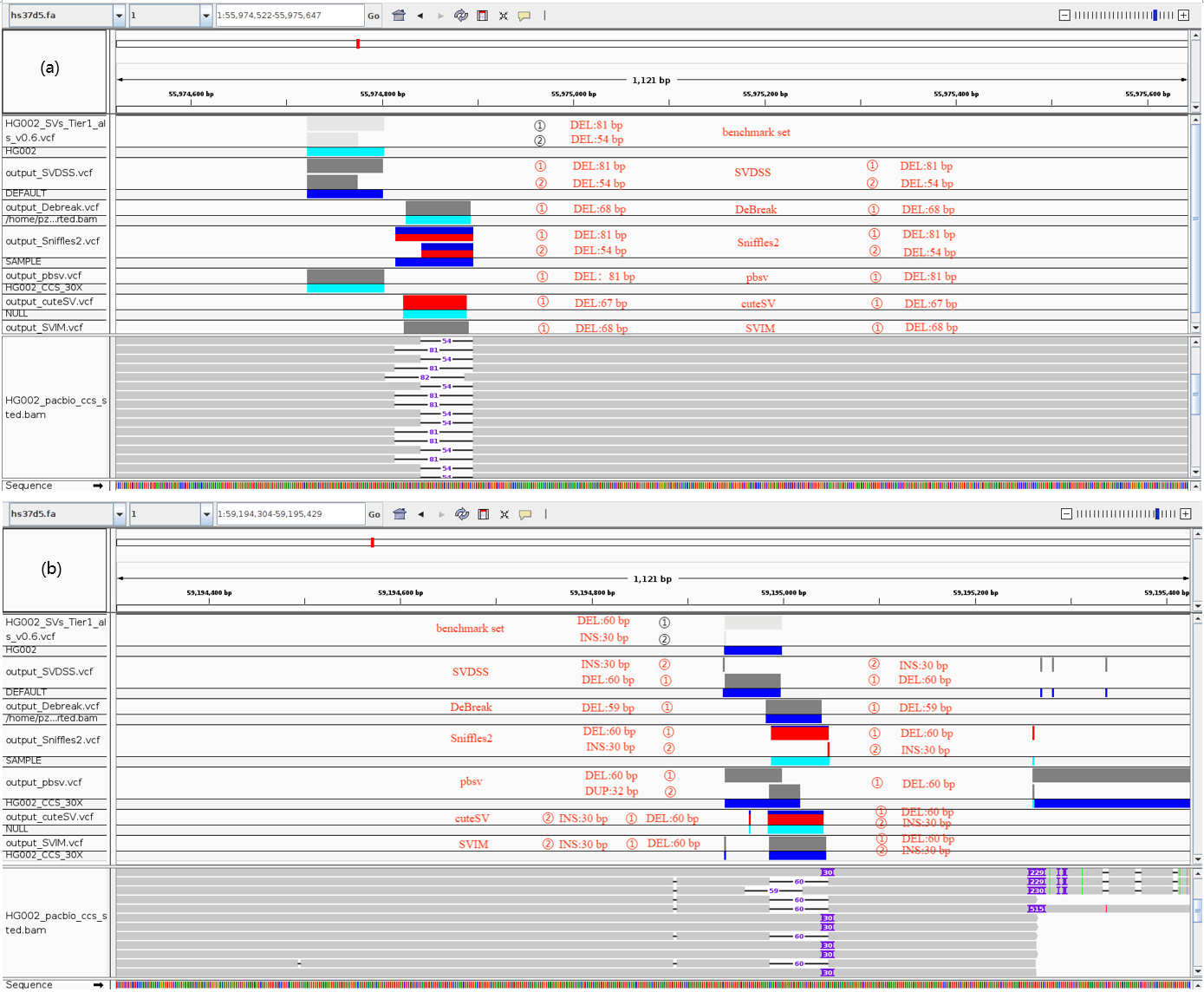


**Supplementary Figure S15. Examples of deletion SV regions with alleles have been accurately matched by ASVBM.** Within the benchmark set, there are two insertions of varying lengths, yet only one or two variants are identified in the detection results. The detection results of different methods are shown, and a comparison of the two variants could be made by matching the corresponding numbers within the red circles. On the left are the ASVBM matching results, and on the right are the Truvari matching results.

In the Tier 1 benchmark set of human chromosome1, regions with multi-allelic variants are analyzed. In Figure S15 (a), at the chr1:55974722-55974722 locus in the benchmark set, allelic variants were observed with deletions of lengths 81 bp and 54 bp. The SVDSS, and Sniffles2 detection methods accurately identified and precisely matched the variant in the benchmark set. However, DeBreak, pbsv, cuteSV, and SVIM identified only one variant, but with inaccurate size recognition. These methods may have limitations when multiple variants exist within a single region, especially when the SVs are close together or have similar features, which may result in reporting only one variant. On the right, Truvari's results are consistent with ASVBM's results. In Figure S15 (b), at the chr1:59194939-59194939 locus in the benchmark set, allelic variants were observed with a 60 bp deletion and a 30 bp insertion. Most detection methods accurately identified both variants, except for DeBreak, which only detected a 59 bp deletion in its detection results. During variant matching, considering the matching of SV types, pbsv exhibited a phenomenon of misclassifying INS as DUP. When considering DUP as INS, both variants were accurately matched to the corresponding items in the benchmark set. On the right, Truvari's results are consistent with ASVBM's results.

**3. Supplementary Tables**

**Supplementary Table S1. SV benchmarking results for multiple user callsets with different matching modes using HG002 PacBio CCS data**

| Option | Tool | SV callers | #TP | # FP | #FN | #LP | Rc | Pr | F1 |
| --- | --- | --- | --- | --- | --- | --- | --- | --- | --- |
| loose match | ASVBM | SVDSS | 38154 | 14142 | 35858 | 2459 | 51.6 | 73.0 | 60.4 |
|  |  | DeBreak | 44908 | **8886** | 29104 | **99** | 60.7 | **83.5** | 70.3 |
|  |  | Sniffles2 | 47362 | 12035 | 26650 | 1228 | 64.0 | 79.7 | **71.0** |
|  |  | pbsv | 45507 | 12275 | 28505 | 718 | 61.5 | 78.8 | 69.1 |
|  |  | cuteSV | 30261 | 11277 | 43751 | 1174 | 40.9 | 72.9 | 52.4 |
|  |  | SVIM | **51510** | 42845 | **22502** | 3230 | **69.6** | 54.6 | 61.2 |
|  | Truvari | SVDSS | 37474 | 15478 | 36404 | N/A | 50.7 | 72.3 | 59.6 |
|  |  | DeBreak | 45392 | **8653** | 28486 | N/A | 61.4 | **83.1** | **70.7** |
|  |  | Sniffles2 | 46871 | 12736 | 27007 | N/A | 63.4 | 77.7 | 69.8 |
|  |  | pbsv | 38706 | 17545 | 35172 | N/A | 52.4 | 67.4 | 58.9 |
|  |  | cuteSV | 29742 | 12048 | 44136 | N/A | 40.3 | 69.7 | 51.1 |
|  |  | SVIM | **50863** | 44581 | **23015** | N/A | **68.8** | 52.1 | 59.3 |
| strict match | ASVBM | SVDSS | 38154 | 14142 | 35858 | 2459 | 51.6 | 73.0 | 60.4 |
|  |  | DeBreak | 44892 | 8902 | 29120 | 99 | 60.7 | 83.5 | 70.3 |
|  |  | Sniffles2 | 47356 | 12043 | 26656 | 1228 | 64.0 | 79.7 | **71.0** |
|  |  | pbsv | 37727 | 19655 | 36285 | 718 | 51.0 | 65.8 | 57.4 |
|  |  | cuteSV | 30217 | **11347** | 43795 | 1174 | 40.8 | **72.7** | 52.3 |
|  |  | SVIM | **51480** | 42934 | **22532** | **3230** | **69.6** | 54.5 | 61.1 |
|  | Truvari | SVDSS | 37474 | 15478 | 36404 | N/A | 50.7 | 72.3 | 59.6 |
|  |  | DeBreak | 45392 | 8653 | 28486 | N/A | 61.4 | 83.1 | **70.7** |
|  |  | Sniffles2 | 46871 | 12736 | 27007 | N/A | 63.4 | 77.7 | 69.8 |
|  |  | pbsv | 37437 | 18806 | 36441 | N/A | 50.7 | 65.0 | 57.0 |
|  |  | cuteSV | 29742 | **12048** | 44136 | N/A | 40.3 | **69.7** | 51.1 |
|  |  | SVIM | **50863** | 44581 | **23015** | N/A | **68.8** | 52.1 | 59.3 |

Note: The benchmark set contains a total of 74,012 variants. Results are shown in the benchmarking results statistics obtained using different type matching criteria by ASVBM and Truvari, loose match allows for matches between duplications and insertions, while strict match indicates strict matches of the same SV type. N/A: not applicable. Rc stands for Recall, Pr stands for Precision, and F1 stands for F1 score.

**Supplementary Table S2. Statistics of true positives validated by local joint variant analysis using HG002 PacBio CCS data**

| Aligners | SV caller | Number of latent positives merged to validate a TP | | | | | | | | | #TPs |
| --- | --- | --- | --- | --- | --- | --- | --- | --- | --- | --- | --- |
|  |  | 2 | 3 | 4 | 5 | 6 | 7 | 8 | 9 | 10 |  |
| minimap2 | SVDSS | 786 | 162 | 37 | 12 | 5 | 3 | 3 | 0 | 0 | 1008 |
|  | DeBreak | 48 | 1 | 0 | 0 | 0 | 0 | 0 | 0 | 0 | 49 |
|  | Sniffles2 | 500 | 61 | 4 | 2 | 0 | 0 | 0 | 0 | 0 | 567 |
|  | pbsv | 315 | 25 | 1 | 0 | 0 | 0 | 0 | 0 | 0 | 341 |
|  | cuteSV | 469 | 61 | 6 | 1 | 0 | 0 | 0 | 0 | 0 | 537 |
|  | svim | 895 | 214 | 84 | 31 | 9 | 3 | 3 | 1 | 0 | 1240 |
| ngmlr | SVDSS | 431 | 33 | 4 | 1 | 1 | 0 | 0 | 0 | 1 | 471 |
|  | DeBreak | 22 | 1 | 0 | 0 | 0 | 0 | 0 | 0 | 0 | 23 |
|  | Sniffles2 | 335 | 23 | 1 | 0 | 0 | 0 | 0 | 0 | 0 | 359 |
|  | pbsv | 201 | 12 | 0 | 0 | 0 | 0 | 0 | 0 | 0 | 213 |
|  | cuteSV | 232 | 12 | 0 | 0 | 0 | 0 | 0 | 0 | 0 | 244 |
|  | svim | 481 | 63 | 10 | 2 | 0 | 0 | 0 | 0 | 0 | 556 |

Note: Multiple smaller variants, when analyzed jointly, can validate a larger variant in the benchmark set and accurately classify it as a TP. These smaller variants are referred to as latent positives (LPs). This table summarizes the number of TPs validated by merging multiple predicted variants (i.e., LPs) from different detection methods. Specifically, it shows how many TPs were confirmed by merging 2, 3, 4, etc., LPs. The concept of latent positives refers to multiple overlapping or adjacent variant calls that collectively support a single TP. For example, 786 TPs were validated by merging two LPs each, resulting in a total of 786 × 2 = 1,572 latent positives; similarly, 162 TPs were validated by merging three LPs each using SVDSS, corresponding to a total of 162 × 3 = 486 latent positives.

**Supplementary Table S3. SV benchmarking results of human genome PacBio CCS data with different SV size ranges**

| Region | Caller | #TP | #FP | #FN | Pr | Rc | F1 | SeqSim |
| --- | --- | --- | --- | --- | --- | --- | --- | --- |
| 1-100 | SVDSS | 24269 | 8860 | 29952 | 74.8 | 44.8 | 56.0 | 97.9 |
|  | DeBreak | 31879 | **6355** | 22342 | **82.7** | 58.8 | 68.7 | 95.9 |
|  | Sniffles2 | 33643 | 7884 | 20578 | 80.2 | 62.0 | **70.0** | 97.4 |
|  | pbsv | 32294 | 7671 | 21927 | 79.9 | 59.6 | 68.2 | **98.8** |
|  | cuteSV | 16488 | 5454 | 37733 | 73.8 | 30.4 | 43.1 | 97.5 |
|  | SVIM | **37629** | 32791 | **16592** | 52.3 | **69.4** | 59.6 | 98.3 |
| 101-250 | SVDSS | 5350 | 2588 | 2123 | 68.1 | 71.6 | 69.8 | **96.8** |
|  | DeBreak | 4935 | **1212** | 2538 | **78.3** | 66.0 | **71.6** | 92.6 |
|  | Sniffles2 | 5248 | 1969 | 2225 | 70.7 | 70.2 | 70.4 | 96.2 |
|  | pbsv | 4977 | 2034 | 2496 | 68.6 | 66.6 | 67.6 | **96.8** |
|  | cuteSV | 5216 | 2433 | 2257 | 65.9 | 69.8 | 67.8 | 96.1 |
|  | SVIM | **5379** | 4870 | **2094** | 49.9 | **72.0** | 58.9 | 96.5 |
| 251-500 | SVDSS | 4490 | 1300 | 1707 | 78.4 | 72.5 | 75.3 | 97.1 |
|  | DeBreak | 4343 | **624** | 1854 | **87.1** | 70.1 | **77.7** | 94.6 |
|  | Sniffles2 | 4464 | 1050 | 1733 | 80.0 | 72.0 | 75.8 | 96.8 |
|  | pbsv | 4302 | 1130 | 1895 | 78.4 | 69.4 | 73.7 | **97.2** |
|  | cuteSV | 4470 | 1381 | 1727 | 75.5 | 72.1 | 73.8 | 96.7 |
|  | SVIM | **4523** | 2468 | **1674** | 63.4 | **73.0** | 67.9 | 96.9 |
| 501-1000 | SVDSS | **1729** | 712 | **785** | 70.7 | **68.8** | 69.7 | **94.6** |
|  | DeBreak | 1638 | **329** | 876 | **82.4** | 65.2 | **72.8** | 90.6 |
|  | Sniffles2 | 1701 | 535 | 813 | 74.1 | 67.7 | 70.7 | 94.1 |
|  | pbsv | 1628 | 674 | 886 | 68.8 | 64.8 | 66.7 | 93.9 |
|  | cuteSV | 1704 | 846 | 810 | 64.8 | 67.8 | 66.3 | 94.0 |
|  | SVIM | **1729** | 1269 | **785** | 54.8 | **68.8** | 61.0 | 94.2 |
| 1-2.5 kbp | SVDSS | **1441** | 442 | **603** | 75.1 | **70.5** | 72.7 | **94.5** |
|  | DeBreak | 1338 | **251** | 706 | **83.2** | 65.5 | **73.3** | 92.7 |
|  | Sniffles2 | 1422 | 372 | 622 | 76.8 | 69.6 | 73.0 | 94.1 |
|  | pbsv | 1383 | 458 | 661 | 72.5 | 67.7 | 70.0 | 94.1 |
|  | cuteSV | 1433 | 610 | 611 | 67.3 | 70.1 | 68.7 | 94.0 |
|  | SVIM | 1399 | 851 | 645 | 58.5 | 68.4 | 63.1 | 94.1 |
| 2.5-5 kbp | SVDSS | 571 | 170 | 250 | 75.9 | 69.5 | 72.6 | 95.8 |
|  | DeBreak | 495 | **80** | 326 | **86.0** | 60.3 | 70.9 | **96.5** |
|  | Sniffles2 | 574 | 155 | 247 | 76.8 | 69.9 | **73.2** | 95.5 |
|  | pbsv | 572 | 174 | 249 | 74.6 | 69.7 | 72.0 | 95.4 |
|  | cuteSV | **584** | 217 | **237** | 70.9 | **71.1** | 71.0 | 95.0 |
|  | SVIM | 536 | 293 | 285 | 61.7 | 65.3 | 63.4 | 95.6 |
| 5-10 kbp | SVDSS | 267 | 58 | 210 | 81.6 | 56.0 | 66.4 | 97.2 |
|  | DeBreak | 229 | **24** | 248 | **90.3** | 48.0 | 62.7 | **97.9** |
|  | Sniffles2 | 263 | 44 | 214 | 84.5 | 55.1 | 66.7 | 97.1 |
|  | pbsv | 299 | 81 | 178 | 77.1 | 62.7 | **69.1** | 96.3 |
|  | cuteSV | **304** | 125 | **173** | 69.0 | **63.7** | 66.3 | 94.6 |
|  | SVIM | 264 | 134 | 213 | 64.5 | 55.3 | 59.6 | 97.1 |
| >10 kbp | SVDSS | 37 | 12 | 228 | 73.9 | 14.0 | 23.5 | 99.6 |
|  | DeBreak | 51 | **11** | 214 | **82.3** | 19.2 | **31.2** | **100** |
|  | Sniffles2 | 47 | 17 | 218 | 72.1 | 17.7 | 28.5 | **100** |
|  | pbsv | 52 | 53 | 213 | 49.5 | 19.6 | 28.1 | 99.3 |
|  | cuteSV | **62** | 211 | **203** | 22.7 | **23.4** | 23.0 | 98.0 |
|  | SVIM | 51 | 169 | 214 | 22.5 | 19.2 | 20.7 | **100** |

Note: The benchmark set contains a total of 74,012 variants. TP: True Positive, which refers to the number of correctly identified targets or events. FP: False Positive, indicating the number of falsely identified targets or events. FN: False Negative, which refers to the number of targets or events that were missed or not identified correctly. Pr: Precision, which refers to the proportion of true positives in the results. Rc: Recall, which represents the number of true positives identified. SeqSim: the measure of sequence similarity, which is calculated for matched SV pairs that include sequences. Rc stands for Recall, Pr stands for Precision, and F1 stands for F1 score.

**Supplementary Table S4. SV benchmarking results of Alleles matching using ASVBM and Truvari**

| Tool | SV callers | #TP | # FP | #FN | #LP | Rc | Pr | F1 | SeqSim |
| --- | --- | --- | --- | --- | --- | --- | --- | --- | --- |
| ASVBM | SVDSS | 3105 | 325 | 2517 | 76 | 55.2 | 90.5 | 68.6 | 95.9 |
|  | DeBreak | 3067 | **145** | 2555 | **8** | 54.6 | **95.5** | 69.4 | 91.2 |
|  | Sniffles2 | 3181 | 214 | 2441 | 20 | 56.6 | 93.7 | 70.6 | 94.4 |
|  | pbsv | 3045 | 201 | 2577 | 14 | 54.2 | 93.8 | 68.7 | 94.9 |
|  | cuteSV | 2673 | 222 | 2949 | 20 | 47.5 | 92.3 | 62.8 | 94.4 |
|  | SVIM | **3475** | 462 | **2147** | 118 | **61.8** | 88.3 | 72.7 | 95.1 |
| Truvari | SVDSS | 3020 | 384 | 2594 | N/A | 53.8 | 88.7 | 67.0 | N/A |
|  | DeBreak | 3127 | **171** | 2487 | N/A | 55.7 | **93.6** | 69.8 | N/A |
|  | Sniffles2 | 3110 | 274 | 2504 | N/A | 55.4 | 90.6 | 68.8 | N/A |
|  | pbsv | 2416 | 704 | 3198 | N/A | 43.0 | 74.1 | 54.4 | N/A |
|  | cuteSV | 2598 | 283 | 3016 | N/A | 46.3 | 88.7 | 60.8 | N/A |
|  | SVIM | **3389** | 559 | **2225** | N/A | **60.4** | 83.8 | 70.2 | N/A |

Note: The benchmark set contains a total of 5,622 variants. SV callers detected variants that were greater than 20 bp in size. TP: True Positive, which refers to the number of correctly identified targets or events. FP: False Positive, indicating the number of falsely identified targets or events. FN: False Negative, which refers to the number of targets or events that were missed or not identified correctly. LP: Latent Positive, partially positives obtained through local joint analysis validation. SeqSim: the measure of sequence similarity, which is calculated for matched SV pairs that include sequences. N/A: not applicable. Rc stands for Recall, Pr stands for Precision, and F1 stands for F1 score.
